# EEG entropy measures in anesthesia

**DOI:** 10.3389/fncom.2015.00016

**Published:** 2015-02-18

**Authors:** Zhenhu Liang, Yinghua Wang, Xue Sun, Duan Li, Logan J. Voss, Jamie W. Sleigh, Satoshi Hagihira, Xiaoli Li

**Affiliations:** ^1^Institute of Electrical Engineering, Yanshan UniversityQinhuangdao, China; ^2^State Key Laboratory of Cognitive Neuroscience and Learning and IDG/McGovern Institute for Brain Research, Beijing Normal UniversityBeijing, China; ^3^Center for Collaboration and Innovation in Brain and Learning Sciences, Beijing Normal UniversityBeijing, China; ^4^Institute of Information Science and Engineering, Yanshan UniversityQinhuangdao, China; ^5^Department of Anesthesia, Waikato HospitalHamilton, New Zealand; ^6^Department of Anesthesiology, Osaka University Graduate School of MedicineOsaka, Japan

**Keywords:** EEG, anesthesia, entropy, pharmacokinetic/pharmacodynamic modeling, depth of anesthesia monitoring

## Abstract

**Highlights:**

► Twelve entropy indices were systematically compared in monitoring depth of anesthesia and detecting burst suppression.
► Renyi permutation entropy performed best in tracking EEG changes associated with different anesthesia states.
► Approximate Entropy and Sample Entropy performed best in detecting burst suppression.

**Objective:** Entropy algorithms have been widely used in analyzing EEG signals during anesthesia. However, a systematic comparison of these entropy algorithms in assessing anesthesia drugs' effect is lacking. In this study, we compare the capability of 12 entropy indices for monitoring depth of anesthesia (DoA) and detecting the burst suppression pattern (BSP), in anesthesia induced by GABAergic agents.

**Methods:** Twelve indices were investigated, namely Response Entropy (RE) and State entropy (SE), three wavelet entropy (WE) measures [Shannon WE (SWE), Tsallis WE (TWE), and Renyi WE (RWE)], Hilbert-Huang spectral entropy (HHSE), approximate entropy (ApEn), sample entropy (SampEn), Fuzzy entropy, and three permutation entropy (PE) measures [Shannon PE (SPE), Tsallis PE (TPE) and Renyi PE (RPE)]. Two EEG data sets from sevoflurane-induced and isoflurane-induced anesthesia respectively were selected to assess the capability of each entropy index in DoA monitoring and BSP detection. To validate the effectiveness of these entropy algorithms, pharmacokinetic/pharmacodynamic (PK/PD) modeling and prediction probability (*Pk*) analysis were applied. The multifractal detrended fluctuation analysis (MDFA) as a non-entropy measure was compared.

**Results:** All the entropy and MDFA indices could track the changes in EEG pattern during different anesthesia states. Three PE measures outperformed the other entropy indices, with less baseline variability, higher coefficient of determination (*R*^2^) and prediction probability, and RPE performed best; ApEn and SampEn discriminated BSP best. Additionally, these entropy measures showed an advantage in computation efficiency compared with MDFA.

**Conclusion:** Each entropy index has its advantages and disadvantages in estimating DoA. Overall, it is suggested that the RPE index was a superior measure. Investigating the advantages and disadvantages of these entropy indices could help improve current clinical indices for monitoring DoA.

## Introduction

In the operating room, general anesthesia is important to guarantee successful surgery and ensure patients' safety and comfort. For anesthesia, the reliable monitoring of anesthetic drug effects on the brain is a clinical concern for anesthesiologists (Monk et al., 2005). The central nervous system (CNS) is the main target of anesthetic drugs. Originated in CNS, the electroencephalogram (EEG) reflects the neural activities of brain, and has been widely used as a surrogate parameter to quantify the anesthetic drug effect (Rampil, 1998; Bruhn et al., 2006; Jameson and Sloan, 2006). However, only limited information can be obtained from the EEG signals purely by waveform observation. With the development of signal processing, various methods have been applied to analyze, identify or detect mental disorders and consciousness mechanisms from EEG signals (Okogbaa et al., 1994; Natarajan et al., 2004; Abásolo et al., 2006), as well as evaluating the effects of anesthesia.

In recent decades, numerous attempts have been made to develop an index for describing anesthetic drug effects on the brain, including zero crossing frequency, spectral edge, wavelet analysis, high-order spectral analysis etc. These studies laid the foundation of commercial EEG-based monitors of depth of anesthesia (DoA), such as BIS (Aspect Medical Systems, Newton, MA) (Bruhn et al., 2006; Ellerkmann et al., 2010) and M-entropy (GE Healthcare, Helsinki, Finland) (Viertiö-Oja et al., 2004; Bruhn et al., 2006). Many of these methods are derived from linear theories. However, various studies have shown that the EEG is a non-stationary signal that exhibits non-linear or chaotic behaviors (Elbert et al., 1994; Pritchard et al., 1995; Zhang et al., 2001; Natarajan et al., 2004). This prompted many researchers to adopt non-linear analysis methods in anesthesia study, for example largest Lyapunov exponent (Fell et al., 1996), Hurst exponent (Alvarez-Ramirez et al., 2008), fractal analysis (Klonowski et al., 2006; Gifani et al., 2007; Liang et al., 2012), detrended fluctuation analysis (DFA) (Jospin et al., 2007; Nguyen-Ky et al., 2010b), recurrence analysis (Huang et al., 2006), and non-linear entropies (Bruhn et al., 2001; Li et al., 2008a). In particular, non-linear entropy methods describing the complexity of EEG signals, have received considerable attention.

The word “entropy” was first proposed as a thermodynamic principle by Clausius (1867). It describes the distribution probability of molecules of gaseous or fluid systems. In 1949, Claude E. Shannon introduced entropy into information theory to describe the distribution of signal components (Shannon and Weaver, 1949). So far, numerous entropy algorithms have been proposed and used to quantify DoA, covering Spectral entropy [which includes Response Entropy (RE) and State entropy (SE)] (Viertiö-Oja et al., 2004; Klockars et al., 2012), Approximate entropy (ApEn) (Bruhn et al., 2000), Sample entropy (SampEn) (Richman and Moorman, 2000), Fuzzy entropy (FuzzyEn) (Chen et al., 2007), Shannon Permutation entropy (SPE) (Li et al., 2008a, 2012), Shannon Wavelet entropy (SWE) (Särkelä et al., 2007), and Hilbert-Huang spectral entropy (HHSE) (Li et al., 2008b).

Spectral Entropy is the method applied in the commercial M-Entropy Module (Viertiö-Oja et al., 2004). It consists of two parameters: Response Entropy (RE) and State Entropy (SE). SE primarily includes the spectrum of the EEG signal from 0.8 to 32 Hz, and RE includes electromyogram activity from 0.8 to 47 Hz (Viertiö-Oja et al., 2004). Shannon Wavelet entropy (SWE) is the Shannon entropy in the wavelet domain, which indicates signal variation at each frequency scale (Rosso et al., 2001). And the Hilbert–Huang spectral entropy (HHSE) is the Shannon entropy based on the Hilbert–Huang transform proposed by Huang et al. (1998). HHSE has been successfully applied to the anesthetic EEG signals (Li et al., 2008b).

The above methods are based on the frequency spectrum. Whereas many entropy methods are based on the time series and phase space analysis. ApEn is an algorithm derived from the Kolmogorov-Sinai entropy (Pincus, 1991). It quantifies the predictability of subsequent amplitude values of a signal. A previous investigation showed that ApEn correlates well with the concentration of desflurane (Bruhn et al., 2000). However, ApEn lacks relative consistency and is highly dependent on data length, SampEn was proposed to overcome ApEn's limitation by removing self-matching and relieving its bias (Richman and Moorman, 2000). SampEn has been used for analyzing EEG signals (Montirosso et al., 2010; Yoo et al., 2012). FuzzyEn was proposed by Chen et al. (2007). It is based on the fuzzy membership functions to define the vectors' similarity, using the soft and continuous boundaries of fuzzy functions to ensure the continuity and the validity of FuzzyEn's definition (Chen et al., 2009). SPE was introduced by Bandt and Pompe (2002). It is a complexity measure based on symbolic dynamics (Bandt and Pompe, 2002). Because of its simple concept and fast computation, SPE has been widely used in EEG signal analysis (Cao et al., 2004; Li et al., 2007, 2008a). Furthermore, its derivatives, multi-scale permutation entropy (Li et al., 2010) and composite permutation entropy index (Olofsen et al., 2008) have been successfully applied to analyze EEG signals during anesthesia.

However, “No one knows what entropy really is, so in a debate you will always have the advantage.” This statement is true for EEG analysis today (Ferenets et al., 2006). Each entropy index has its own advantages and disadvantages, but how does their performance compare when evaluating the effect of anesthesia on brain activity? To this end, some researchers have compared the performance of different entropy methods for anesthesia monitoring (Sleigh et al., 2001, 2005; Bein, 2006). Unfortunately, these articles analyzed no more than three entropies. To our knowledge, a systematic comparison of the performance of them in assessing anesthesia drug effect is lacking. In this study, we aim to compare the capability of several commonly used entropy indices for monitoring DoA.

We noticed that definitions of all the above entropies are based on Shannon information theory, which belongs to a short-range or extensive concept. However, the physical systems especially the biomedical systems are often characterized by either long-range interactions, long-term memories, or multifractality (Zunino et al., 2008). To describe these characters, two generalized forms of entropy were proposed: Renyi entropy (Renyi, 1970) and Tsallis entropy (*q*-entropy) (Tsallis et al., 1998). For example Tsallis entropy has a parameter *q* for non-extensity. If *q* > 1, the entropy is more sensitive to events that occur often, whereas if 0 < *q* < 1 it is more sensitive to the events that occur seldom (Maszczyk and Duch, 2008). In the limit *q* → 1, it coincides with Shannon entropy. These generalized entropies can provide additional informational about the importance of specific events, such as outliers or rare events. The two classes of entropies and their combinations with current signal processing methods have been already applied in EEG analysis (Bezerianos et al., 2003; Tong et al., 2003; Inuso et al., 2007) and often been proved advantageous than the Shannon version (Zunino et al., 2008; Arefian et al., 2009). To make the research more instructive, we believe it useful to investigate these non-extensive entropy measures along with those extensive Shannon entropies in DoA monitoring. In this study, we involved the Tsallis wavelet entropy (TWE) and Renyi wavelet entropy (RWE) proposed by Rosso et al. (2003, 2006), as well as the Tsallis permutation entropy (TPE) proposed by Zunino et al. (2008) and a new Renyi permutation entropy (RPE).

For illustrative purpose, we divide the entropies into two families:

1. Entropies in the time-frequency domain: RE, SE, SWE, TWE, RWE, and HHSE;
2. Entropies in the time domain: ApEn, SampEn, FuzzyEn, SPE, TPE, and RPE.

In this work, their performance for monitoring DoA were compared. Using data sets obtained during sevoflurane and isoflurane anesthesia, we quantified for each index the responsiveness to loss of consciousness, computation complexity and the ability to detect BSP. Pharmacokinetic/pharmacodynamic (PK/PD) modeling and prediction probability statistics were applied to evaluate the efficiency of each index for tracking anesthetic concentration. Additionally, in order to prove the efficiency of the entropy approaches, two non-linear dynamic methods: DFA (Jospin et al., 2007) and multifractal DFA (MDFA) (Kantelhardt et al., 2002) are compared.

## Entropy indices

The computation of each entropy index is briefly described as follows.

### Spectral entropy (RE and SE)

Spectral Entropy quantifies the probability density function (PDF) of the signal power spectrum in the frequency domain. The detail of the Spectral Entropy algorithm can be seen in Inouye et al. (1991) and Rezek and Roberts (1998). Spectral Entropy consists of the RE and the SE. RE is computed over a frequency range from 0.8 to 47 Hz while SE is computed over the frequency range from 0.8 to 32 Hz. The normalization step for RE and SE are defined as follows:

(1) $$RE=\frac{H_{sp_{0.8-47}}}{\log(N_{0.8-47})}$$

(2) $$SE=\frac{H_{sp_{0.8-32}}}{\log(N_{0.8-47})}$$

where *H*_*sp*_0.8−47__ and *H*_*sp*_0.8−32__ means the sum of spectral power between 0.8 and 47 Hz, and 0.8 to 32 Hz, respectively. And *N*_0.8−47_ equals the total number of frequency components in the range 0.8–47 Hz. Spectral Entropy describes the degree of skewness in the frequency distribution. For example, in the normalized case, the Spectral Entropy of a pure sine wave with a single spectral peak is 0, while that of white noise is 1.

### Wavelet entropy (SWE, TWE, and RWE)

WE differentiates specific brain states under spontaneous or stimulus-related conditions and recognizes the time localizations of a dynamic process. To calculate Wavelet Entropy, wavelet energy *E*_*j*_ of a signal is determined at each scale *j* as follows:

(3) $$E_{j}=\sum_{k\;=\;1}^{L_{j}}d{\left(k\right)}^{2}$$

where *k* and *L*_*j*_ are the summation index and the number of coefficients at each scale *j* with in a given epoch, respectively. The total energy over all scales is obtained by:

(4) $$E_{total}=\sum_{j}E_{j}=\sum_{j}\sum_{k\;=\;1}^{L_{j}}d_{j}{\left(k\right)}^{2}$$

Then wavelet energy is divided by total energy to obtain the relative wavelet energy at each scale *j*:

(5) $$p_{j}=\frac{E_{j}}{E_{total}}=\frac{E_{j}}{\sum_{j}E_{j}}=\frac{\sum_{k\;=\;1}^{L_{j}}d{\left(k\right)}^{2}}{\sum_{j}\sum_{k\;=\;1}^{L_{j}}d_{j}{\left(k\right)}^{2}}$$

SWE is calculated from Shannon entropy of *p*_*j*_ distribution between scales as follows:

(6) $$S^{\left(s\right)}=-\sum_{j}p_{j}\log p_{j}$$

The detail of the algorithm used in this study can be seen in Särkelä et al. (2007).

And the TWE is defined as,

(7) $$S_{q}^{\left(T\right)}=\frac{1}{q-1}\sum_{j}\left[p_{j}-{\left(p_{j}\right)}^{q}\right]$$

where *q* is a non-extensity parameter.

Based on the definition of Renyi entropy (Renyi, 1970), the RWE is defined as Rosso et al. (2006):

(8) $$S_{a}^{\left(R\right)}=\frac{1}{1-a}log\left[\sum_{j}{\left(p_{j}\right)}^{a}\right]$$

For *S*^(*S*)^_*q*_, the normalized SWE is

(9) $$SWE=S^{\left(s\right)}/\log N_{J}$$

where *N*_*J*_ is the number of wavelet resolution levels.

And *S*^(*T*)^_*q*_ is normalized by dividing [1 − *N*^1 − *q*^_*J*_]/(*q* − 1), defined by Rosso et al. (2003):

(10) $$TWE=\frac{S_{q}^{\left(T\right)}}{\left[1-N_{J}^{1\;-\;q}\right]/(q-1)}$$

Further, the normalized *S*^(*R*)^_*a*_ is defined as Maszczyk and Duch (2008):

(11) $$RWE=\frac{S_{a}^{\left(R\right)}}{\log N_{J}}$$

The values of three WE measures depend on the wavelet basis function, the number of decomposed layers (*n*) and the data length (*N*). Furthermore, the TWE and RWE are related to the parameters *q* and *a* respectively. Among these parameters, the wavelet basis function is most important. Because of the lack of a fixed criterion, it is very difficult to select an appropriate wavelet basis function in practical applications and many studies choose it based on experiments. The details of the selection process in this study can be found in Supplement Material 1.

### Hilbert-Huang spectral entropy (HHSE)

HHSE is based on the Hilbert-Huang transform, which applies the Shannon entropy concept to the Hilbert-Huang spectrum. The detail of the algorithm is seen in Li et al. (2008b). For a given non-stationary signal *x*(*t*), the EMD method decomposes the signal into a series of intrinsic mode functions (IMFs), *C*_*n*_(1, 2, …, *M*), where *M* is the number of IMFs. The signal *x*(*t*) can be written by:

(12) $$x\left(t\right)=\sum_{i\;=\;1}^{n\;-\;1}imf{\left(t\right)}_{i}+r_{n}\left(t\right)$$

Apply the Hilbert transform to the IMF components,

(13) $$Z\left(t\right)=imf\left(t\right)+iH\left[imf(t)\right]=a\left(t\right)e^{i\int \omega \left(t\right)dt}$$

in which $a\left(t\right)=\sqrt{imf^{2}\left(t\right)+H^{2}\left[imf(t)\right]}$, $\omega \left(t\right)=\frac{d}{dt}\left[\arctan(H\left[imf(t)\right]/imf(t))\right]$, where ω(*t*) and *a*(*t*) are the instantaneous frequency and amplitude, respectively, of the IMFs.

The Hilbert-Huang marginal spectrum is defined by:

(14) h(ω)=∫H(ω,t)dt

To simplify the representation, the Hilbert-Huang spectrum is denoted as a function of frequency (*f*) instead of angular frequency (ω). The marginal spectrum is normalized by:

(15) $$\hat{h}\left(f\right)=h(f)/\sum h(f)$$

Next, the Shannon entropy concept is applied to the Hilbert-Huang spectrum, and Hilbert-Huang spectral entropy is obtained by:

(16) $$HHSE=-\sum_{f}\hat{h}\left(f\right)log\left(\hat{h}\left(f\right)\right)$$

The HHSE values are mainly affected by the frequency resolution and data length (*N*). For accurate computation, the frequency resolution is chosen as 0.1 Hz. *N* directly influences the EMD. In general, the boundary effect may be induced if *N* is too large or too small, which can contaminate the data and distort the power spectrum. The selection of *N* in this study is given in Supplement Material 1.

### Approximate entropy (ApEn)

ApEn is derived from Kolmogorov entropy. It was introduced by Pincus (1991). It can be used to analyze a finite length signal and describe its unpredictability or randomness. Its computation involves embedding the signal into the phase space and estimating the rate of increment in the number of phase space patterns within a predefined value *r*, when the embedding dimension of phase space increases from *m* to *m* + 1.

For a time series *x*(*i*), 1 ≤ *i* ≤ *N* of finite length *N*, reconstitute the *N* − *m* + 1 vectors *X*_*m*_(*i*) following the form:

(17) $$\begin{array}{l} X_{m}\left(i\right)=\left\{x\left(i\right),x\left(i+1\right),\ldots ,x(i+m-1)\right\},\\ \;i=1,2,\ldots ,N-m+1 \end{array}$$

where *m* is the embedding dimension.

Let *C*^*m*^_*i*_(*r*) be the probability that any vector *X*_*m*_(*j*) is within distance *r* of *X*_*m*_(*i*), defined as:

(18) $$\begin{array}{l} C_{i}^{m}\left(r\right)=\frac{1}{N-m+1}\sum_{j\;=\;1}^{N-m+1}\Theta \left(d_{ij}^{m}-r\right);\\ \;i,j=1,2,\ldots ,N-m+1 \end{array}$$

where d is the distance between the vectors *X*_*m*_(*i*) and *X*_*m*_(*j*), defined as:

(19) $$\begin{array}{l} d_{ij}^{m}=d\left[X_{i}^{m},X_{j}^{m}\right]=\max\left(\left|x\left(i+k\right)-x(j-k)\right|\right),\\ \;k=0,1,\ldots ,m \end{array}$$

and Θ is the Heaviside function.

After that, define a parameter Φ^*m*^(*r*):

(20) $$\Phi^{m}\left(r\right)={\left(N-m+1\right)}^{-1}\sum_{i\;=\;1}^{N\;-\;m\;+\;1}\ln C_{i}^{m}\left(r\right)$$

Next, when the dimension changes to *m* + 1, the above process is repeated.

(21) $$\Phi^{m\;+\;1}\left(r\right)={\left(N-m\right)}^{-1}\sum_{i\;=\;1}^{N\;-\;m}\ln C_{i}^{m\;+\;1}\left(r\right)$$

Finally, the approximate entropy is defined by:

(22) $$ApEn\left(m,r,N\right)=\Phi^{m}\left(r\right)-\Phi^{m\;+\;1}(r)$$

The detailed algorithm is seen in Bruhn et al. (2000). The ApEn index is influenced by data length (*N*), tolerance (*r*) and embedding dimension (*m*). According to Pincus (1991) and Bruhn et al. (2000), *N* is recommended to be 1000, *r* 0.1~0.25 of the standard deviation of the signal and *m* 2~3. The selection of these parameters is described in Supplement Material 1.

### Sample entropy (SampEn)

The SampEn proposed by Richman and Moorman (2000) is based on ApEn but differs from it in three ways to remove bias:

1. SampEn eliminates self-matches;
2. To avoid ln 0 caused by removing self-matches, SampEn computes the additional operation of the total number of template well-matches prior to the logarithmic operation.
3. In order to have an equal number of patterns for both embedding dimension *m* and *m* + 1, the time series reconstitution in SampEn have *N* − *m* rows instead of *N* − *m* + 1 in ApEn in embedding dimension *m*.

The first step of calculating SampEn is the same as ApEn. When the embedding dimension is *m*, the total number of template matches is:

(23) $$B^{m}(r)={\left(N-m\right)}^{-1}\sum_{i\;=\;1}^{N\;-\;m}C_{i}^{m}\left(r\right)$$

Similarly, when the embedding dimension is *m* + 1, the total number of template matches is:

(24) $$A^{m}(r)={\left(N-m\right)}^{-1}\sum_{i\;=\;1}^{N\;-\;m}C_{i}^{m\;+\;1}\left(r\right)$$

Finally, the SampEn of the time series is estimated by:

(25) $$\text{SampEn}\left(\text{r},\text{m},\text{N}\right)=-\ln\frac{{\text{A}}^{\text{m}}(\text{r})}{{\text{B}}^{\text{m}}(\text{r})}$$

SampEn is based on ApEn, so its parameter selection procedure is similar to that of ApEn (see Supplement Material 1).

### Fuzzy entropy (FuzzyEn)

Zadeh introduced the concept of “fuzzy set” (Zadeh, 1965). Fuzzy set provides a mechanism for measuring the degree to which a pattern belongs to a given class, by introducing the concept of “membership degree” having a fuzzy function *u*_*c*_(*x*). The nearer the value *u*_*c*_(*x*) is to unity, the higher the membership grade of *x* in the set *C* will be. Inspired by this, Chen et al. (2007) developed the FuzzyEn based on SampEn. FuzzyEn uses the fuzzy membership function *u*(*d*^*m*^_*ij*_, *r*) to obtain the similarity between *X*^*m*^_*i*_ and *X*^*m*^_*j*_ instead of the Heaviside function.

FuzzyEn is based on SampEn, so its parameter selection is similar to that of SampEn (see Supplement Material 1).

### Permutation entropy (SPE, TPE, and RPE)

There are three types of PE measures involved in this study. PE is an ordinal analysis method, in which a given time series is divided into a series of ordinal patterns for describing the order relations between the present and a fixed number of equidistant past values (Bandt, 2005). The advantage of this method is its simplicity, robustness and low computational complexity (Li et al., 2007).

For an *N*-point normalized time series {*x*(*i*) : 1 ≤ *i* ≤ *N*}, firstly the time series is reconstructed:

(26) $$\begin{array}{l} X_{i}=\{x(i),x(i+\tau ),\ldots ,x(i+(m-1)\tau )\},\\ \;i=1,\;2,\ldots ,N-(m-1)\tau \end{array}$$

where τ is the time delay, *m* is the embedding dimension.

Then, rearrange *X*_*i*_ in an increasing order:

(27) $$\begin{array}{l} \{x\left(i+\left(j_{1}-1\right)\tau \right)\leq x\left(i+\left(j_{2}-1\right)\tau \right))\leq \cdots \\ \;\leq x\left(i+(j_{m}-1\right)\tau \} \end{array}$$

There are *m*! permutations for *m* dimensions. Each vector *X*_*i*_ can be mapped to one of the *m*! permutations.

Next, the probability of the *j*th permutation occurring *p*_*j*_ can be defined as:

(28) $$p_{j}=\frac{n_{j}}{\sum_{j\;=\;1}^{m!}n_{j}}$$

where *n*_*j*_ is the number of times the *j*th permutation occurs.

Based on the probability of the *j*th permutation *p*_*j*_, we define SPE, TPE and RPE as follows.

SPE is just the Shannon entropy associated with the probability distribution *p*_*j*_:

(29) $$S_{1}^{\left(s\right)}=-\sum_{j\;=\;1}^{m!}p_{j}\log p_{j}$$

And the normalized SPE is:

(30) $$SPE_{n}=\frac{S_{1}^{\left(S\right)}}{S_{1,\text{max}}^{\left(s\right)}}=\frac{\sum_{j\;=\;1}^{m!}p_{j}\log p_{j}}{\log(m!)}$$

Based on the definition of Tsallis entropy, Zunino et al., proposed the normalized TPE and defined it as Zunino et al. (2008):

(31) $$TPE=\frac{\sum_{j\;=\;1}^{m!}\left(p_{j}-p_{j}^{q}\right)}{1-{\left(m!\right)}^{1\;-\;q}}$$

Furthermore, the normalized RPE measure based on the Renyi entropy and permutation probability distribution *p*_*j*_ is:

(32) $$RPE_{n}=\frac{\log\sum_{j\;=\;1}^{m!}p_{j}^{a}}{\left(1-a\right)\ln m!}$$

In Li et al. (2008a, 2010, 2012), SPE was used to evaluate the effect of sevoflurane and isoflurane anesthesia on the brain. In this study, the parameters of *m* = 6 and τ = 1 are selected for sevoflurane anesthesia as proposed in Li et al. (2008a). The SPE's parameters for isoflurane anesthesia are the same as those proposed by Li et al. (2012). TPE and RPE are first used in DoA measure, therefore selection of the appropriate parameters of TPE and RPE should be based on the experiments. The details of the selection process is shown in Supplement Material 1.

## Materials and statistical methods

### Subjects and EEG recordings

#### EEG data set during sevoflurane-induced anesthesia

In this study, the first data set we used was from a previous study (McKay et al., 2006), in which 19 patients aged 18–63 years were recruited from Waikato Hospital, Hamilton, New Zealand. The subjects were scheduled for elective gynecologic, general, or orthopedic surgery. All patients fasted for at least 6 h before anesthesia and received no premedication. Patients were American Society of Anesthesiologists physical status I or II and signed written informed consent following approval by the Waikato Hospital ethics committee.

Before application of Ag/AgCl electrodes, the skin was carefully cleaned with an alcohol swab to ensure electrode-skin impedance of less than 7.5 kΩ. A composite electrode, the Entropy™ Sensor, composed of a self-adhering flexible band holding three electrodes were used to record the EEG signals between the forehead and temple (active = FpZ, earth = Fp1, and reference = F8). RE and SE were measured every 5 s with a plug-in M-Entropy S/5 Module (Datex-Ohmeda). The sevoflurane concentration was measured at the mouth at 100/s (McKay et al., 2006). All data were recorded and stored on a laptop computer. Off-line analysis was performed using the MATLAB (version 8, MathWorks Inc.) software.

#### EEG data set during isoflurane-induced anesthesia

The second data set contains 29 patients (9 men and 20 women, age 33–77 year) receiving elective abdominal surgery during combined isoflurane general anesthesia and epidural anesthesia (Hagihira et al., 2002). These patients had no neurologic or psychiatric disorders and didn't receive medication with any drugs known to influence anesthesia. The data recordings were approved by the Osaka Prefectural Habikino Hospital and all patients gave written informed consent.

Each patient was injected intramuscularly with 0.5 mg atropine before entering the operating room. Initially, an epidural catheter was placed at the appropriate spinal location. Then, after confirming the effect of epidural analgesia, 3 mg/kg thiopental was used to induce anesthesia. Anesthesia was subsequently maintained with isoflurane, oxygen, and nitrogen after tracheal intubation. Vecuronium was given as required. Lidocaine 1% (80–110 mg/h; initial dose, 90–100 mg) was administered epidurally. Patients received controlled ventilation to maintain adequate oxygenation and normocapnia. To keep mean blood pressure at 60 mmHg, dopamines were administered as required at a dose of 2–5 μg/(kg·min).

Before induction of anesthesia, five EEG electrodes (A1, A2, FP1, FP2, and FPz) were attached to the patients according to the International 10–20 System. FPz was used as the ground electrode. The EEG signal used was recorded from a unipolar lead (FP1-A1) through a 514 X-2 EEG telemetry system (GE Marquette, Tokyo, Japan) with sample frequency of 512 Hz (another Fp2-A2 channel was not analyzed). Isoflurane was initially increased to 1.5% and then stepped down to 0.7%. The end-tidal concentration of isoflurane was purposely maintained at set levels (1.5, 1.3, 1.1, 0.9, and 0.7%) for 30 min at each level. The EEG recordings at 0.3 and 0.5% isoflurane were collected immediately after the operation. The concentration of isoflurane was continuously monitored and recorded by Canomac (Datex, Helsinki, Finland). The BSP was evident in six of the 29 EEG recordings.

The two data sets used can be obtained by asking the authors of corresponding original papers.

### EEG preprocessing

All the EEG recordings were preprocessed by following the steps outlined in Li et al. (2010) before further analysis. Firstly, data points whose amplitude values exceeded a threshold determined by mean and standard deviation (SD) statistics were removed as outliers. Then, the filter function filter.m was used to remove the frequency components higher than 60 Hz. This FIR filter ensures that phase information is not distorted. Thirdly the stationary wavelet transform was used to reduce electro-oculogram (EOG) artifact. Finally, an inverse filter was used to detect and remove EMG and other high-amplitude transient artifacts.

### Pharmacokinetic/pharmacodynamic modeling

To derive the relationship between effect-site anesthetic drug concentration and the measured EEG index, PK/PD modeling was used. These methods have been successfully used to evaluate the proposed EEG indices (Li et al., 2008a; Olofsen et al., 2008). It describes the relationship between drug dose and its effect through two successive physiological processes (McKay et al., 2006). The pharmacokinetic (PK) side of the model describes the changes in blood concentration of the drug over time, while the pharmacodynamic (PD) aspect shows the relation between the concentration of drug at its effect site and its measured effect. The simplest effect site model is a first order model, defined as:

(33) $$dC_{\text{eff}}/dt=k_{\text{eo}}(C_{\text{et}}-C_{\text{eff}})$$

where *C*_eff_ denotes the effect-site concentration, *k*_eo_ is the first-order rate constant for efflux from the effect compartment and *C*_et_ is the end-tidal concentration.

In addition, a non-linear inhibitory sigmoid *E*_max_ model was used to describe the relationship between the estimated *C*_eff_ and the measured EEG indices.

(34) $$\text{Effect}=E_{\text{max}}-(E_{\text{max}}-E_{\text{min}})\times \frac{C_{\text{eff}}^{\gamma }}{EC_{50}^{\gamma }+C_{\text{eff}}^{\gamma }}$$

where Effect is the processed EEG measure, *E*_max_ and *E*_min_ respectively are the maximum and minimum Effect for each individual, *EC*^γ^_50_ is the drug concentration that causes 50% of the maximum Effect and γ is the slope of the concentration–response relationship.

The coefficient of determination *R*^2^ is calculated by:

(35) $$R^{2}=1-\frac{\sum_{i\;=\;1}^{n}{\left(y_{i}-{\hat{y}}_{i}\right)}^{2}}{\sum_{i\;=\;1}^{n}{\left(y_{i}-\bar{y}\right)}^{2}}$$

where *y*_*i*_ is the measured Effect for a given time and ŷ_*i*_ is corresponding modeled Effect.

*C*_eff_ is estimated by iteratively running the above model with a series of *k*_eo_ values, with the optimal *k*_eo_ yielding the greatest *R*^2^ for each patient.

### MDFA exponent

Kantelhardt et al., proposed the MDFA method to describe the non-stationary time series, which is based on a generalization DFA method (Kantelhardt et al., 2002). Nguyen-Ky et al., used the moving-average DFA method to monitoring the DoA and the results showed that DFA could accurately estimate a patient's hypnotic state (Nguyen-Ky et al., 2010a).

For a time series *x*(*t*) of length *N*, the main computation procedure of MDFA consists of three steps.

Step 1. Construct the profile as the equation showed below,

(36) $$y\left(j\right)=\sum_{i=}^{j}\left[x\left(i\right)-\langle x\rangle \right]$$

where 〈*x*〉 represents the average value of *x*(*t*).

Step 2. Divide the new profile {*y*(*j*)} into *N*_*s*_ = *N*/*s* non-overlapping segments of equal length s. Since the record length *N* may not be a multiple of the considered time scale *s*, a short part at the end of the profile will remain in most cases. In order not to disregard this part of record, the same procedure is repeated starting from the other end of the profile {*y*(*j*)}. Thus, 2*N*_*s*_ segments are obtained altogether.

Step 3. Calculate the local trend for each segment by a least-square fit of the data and calculate the variance *F*^2^(*s*, *v*). Thus, the qth order fluctuation function is calculated as follows:

(37) $$F_{q}\left(s\right)={\left\{\frac{1}{2N_{s}}\sum_{v\;=\;1}^{2N}{[F^{2}\left(s,v\right)}^{q/2}\right\}}^{1/q}$$

If *q* = 0, then

(38) $$F_{0}\left(s\right)=\exp\left\{\frac{1}{4N_{s}}\sum_{v\;=\;1}^{2N_{s}}\ln\left[F^{2}(s,v)\right]\right\}$$

It is obvious that when *q* = 2, we have the standard DFA procedure.

MFDFA characterizes the evolution of *F*_*q*_(*s*) is a function of the segment length *s*. Modeling fluctuations that present a power-law behavior between *F*_*q*_(*s*) and *s*, *F*_*q*_(*s*) ∝ *s*^*h*(*q*)^, where the *h*(*q*) is generalized Hurst exponent.

For the multifractal time series, the scaling behavior is sensitive with the parameter *q*. For positive *q*, *h*(*q*) describes the scaling behavior of the segments with large fluctuations. On the contrary, for negative *q*, *h*(*q*) is sensitive to small fluctuations. For more detail of the MDFA method, see in Kantelhardt et al. (2002).

In this study, we only considered the influence of q with the MDFA measure. The selection of parameter is described in Supplement Material 1.

### Statistical analysis

To further evaluate the correlation between the measured EEG index and underlying anesthetic drug effect, prediction probability (*P*_*k*_) statistics were applied, as described in Smith et al. (1996). Given two random data points with different *C*_eff_, *P*_*k*_ describes the probability that the measured EEG index correctly predicts the *C*_eff_ of the two points. Its definition is:

(39) $$P_{k}=\frac{P_{c}+P_{tx}/2}{P_{c}+P_{d}+P_{tx}}$$

where *P*_*c*_, *P*_*d*_ and *P*_*tx*_ separate the probability that two data points drawn at random, independently and with replacement from the population are a concordance, a discordance or an x-only tie. A value of 1 means that the EEG index is perfectly concordant with *C*_eff_; whereas a value of 0.5 means the EEG index is obtained by chance. When the monotonic relation between the drug concentration and the EEG index is negative, the resultant *P*_*k*_ value is replaced by 1 − *P*_*k*_.

In addition, The Kolmogorov–Smirnov test was used to determine whether the data sets were normally distributed. To assess the index stability during the awake state and the sensitivity to the induction process, the relative coefficient of variation (CV) (Li et al., 2008a) was used. Kruskal-Wallis test was used to determine the significant difference of the index values between awake, induction, anesthesia and recovery states.

## Results

**Figure 1:**
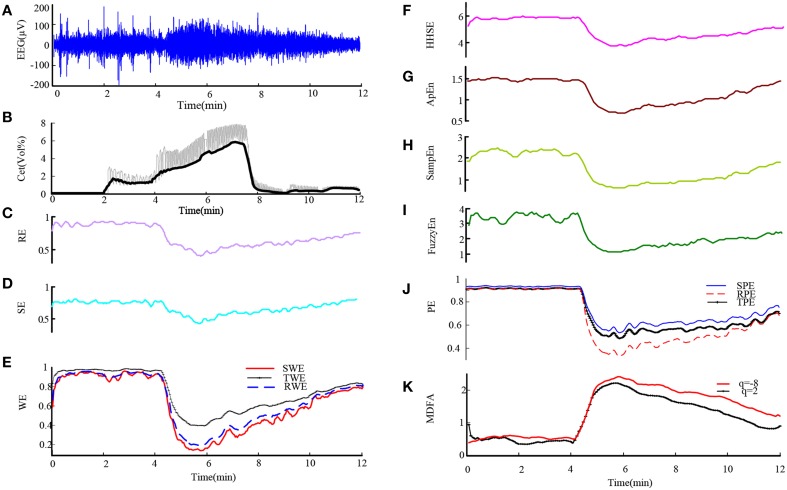
**An EEG recording from a patient undergoing sevoflurane anesthesia and corresponding entropy indices vs. time. (A)** Preprocessed EEG recording. **(B)** Sevoflurane concentration recorded at the mouth (gray line) and the derived end-tidal sevoflurane concentration (black line). **(C–J)** The time course of the studied EEG derivative. The indices are calculated over a window of 10 s with an overlap of 75%. **(K)** The time course of MDFA at *q* = 2 [MDFA(2)] and *q* = −8 [MDFA(−8)]. The window and overlap selection are similar with entropy measures.

First we used these entropy measures on EEG data from sevoflurane anesthesia. Figure 1A shows a preprocessed EEG recording and the derivative from the EEG signal during the whole sevoflurane induction process, from awake to induction, then to deep anesthesia, and finally to recovery. With deepening anesthesia, the mean amplitude of the EEG gradually increased and then the amplitude decreased in the state of recovery. The concurrent end-tidal sevoflurane concentration is represented by the black line given in Figure 1B. It can be regarded as the drug concentration in blood, derived from the recorded sevoflurane concentration at the mouth (represented by gray line). The changes in RE, SE, SWE, TWE, RWE, HHSE, ApEn, SampEn, FuzzyEn, SPE, TPE, RPE, and MDFA corresponding to the EEG recording are successively given in Figures 1C–K. As can be seen, all the entropy indices generally followed the changes in EEG pattern as the drug concentration increased and decreased. And MDFA had the opposite trend with entropy indices.

**Figure 2:**
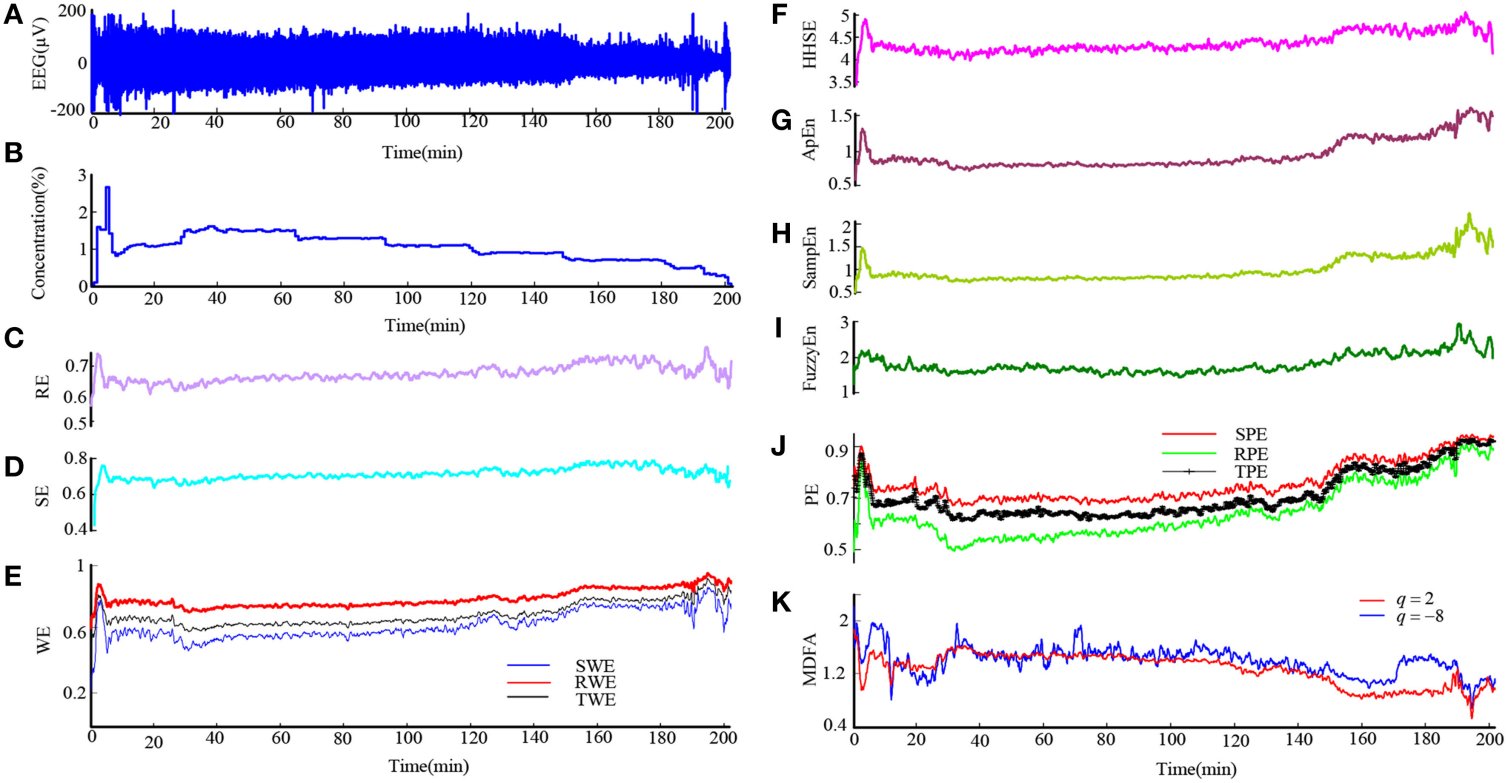
**An EEG recording from a patient in isoflurane anesthesia and calculated indices. (A)** Preprocessed EEG recording, re-sampled at 128 Hz. **(B)** Recording of the isoflurane end-tidal concentration. **(C–J)** Time course of entropy indices, with a time interval of 10 s and 5 s overlap. **(K)** Time course of MDFA measures with a time interval of 10 and 5 s overlap.

Then we analyzed the EEG recording during isoflurane anesthesia using the same entropy algorithms and MDFA methods. Figures 2A,B are the EEG recording and isoflurane end-tidal concentration respectively. It can be seen that the drug concentration increased and then decreased. Figures 2C–K shows the same entropy and MDFA indices as Figures 1C–K, and demonstrate equivalent trends, in line with changes in drug concentration.

**Figure 3:**
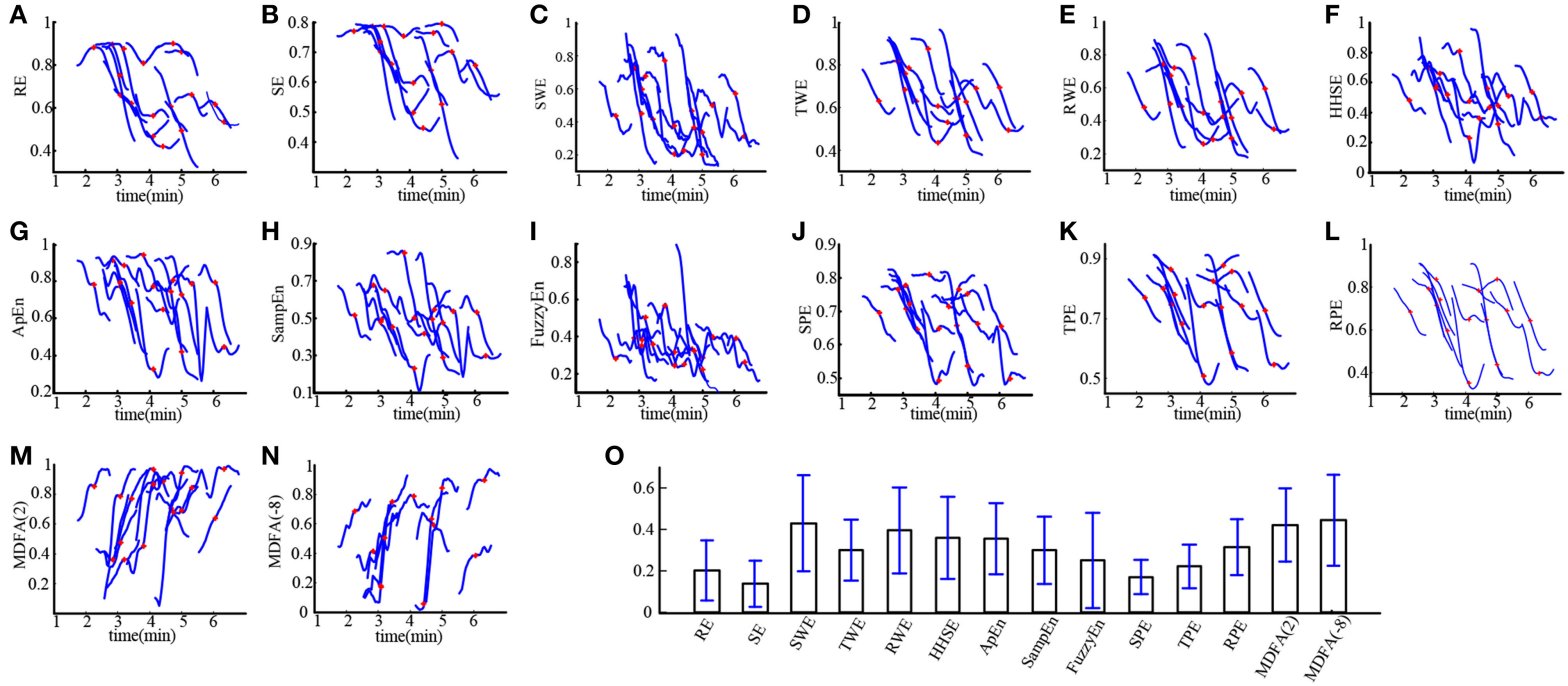
**Entropy and MDFA analysis around the time of LOC for subject undergoing sevoflurane anesthesia (*n* = 19). (A–N)** The normalized indices around LOC (from LOC − 30 s to LOC + 30 s) for all subjects. The red plus sign denotes the point of LOC. **(O)** Statistical analysis of the absolute slope of the linear-fitted polynomials vs. time for studied indices. Bar height indicates the mean value, and the lower and upper line are the 95% confidence interval of each index.

Loss of consciousness (LOC) is the most important clinical time point during anesthesia. We investigated the ability of these entropies in tracking LOC. Figure 3 demonstrates the changes in each index around LOC, from LOC−30 s to LOC+30 s for all subjects during sevoflurane anesthesia. For these plots, index values were normalized to between 0 and 1. It can be seen in Figures 3A–N that MDFA(−8) decreased most rapidly, followed by SWE. Thus, the MDFA with *q* = −8 appeared to be the most sensitive to LOC. To verify this, we calculated the absolute slope values (mean ± SD) of the linear-fitted polynomials vs. time for these indices, as shown in Figure 3O. As can be seen, the absolute slope value for MDFA(−8) (0.44 ± 0.22) is largest, followed by SWE (0.43 ± 0.23).

**Figure 4:**
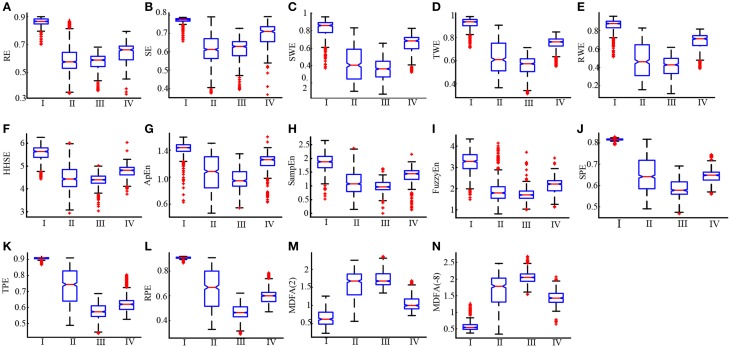
**Box plots of RE, SE, SWE, TWE, RWE, HHEn, ApEn, SampEn, FuzzyEn, SPE, TPE, RPE, MDFA(2) and MDFA(-8) **(A–N)** at awake (I), induction (II), deep anesthesia (III) and recovery (IV) states**.

To further compare the ability of the indices to distinguish different anesthesia states, the sevoflurane anesthesia procedure was divided into four states, i.e., awake, induction, deep anesthesia, and recovery. For each index, a box plot is given in Figure 4. The data was not normally distributed, so the statistics of the 19 patients undergoing sevoflurane anesthesia were expressed as median (min—max), as shown in Table 1. All the entropy indices monotonically decreased as anesthesia deepened, then increased during recovery. The MDFA indices have an opposite trend with the entropy measures. These are consistent with the results in Figure 1. The overlap of three types of PE (SPE, TPE, and RPE) values between the awake and deep anesthesia states were smaller than the other indices. This means the PE has a better ability to separate these states and a greater robustness for individual differences.

**Table 1:** **The statistics of the studied indices at different anesthetic states [median (min-max)]**.

	**Awake**	**Induction**	**Deep anesthesia**	**RoC**
RE	0.87 (0.65–0.90)	0.58 (0.35–0.89)	0.59 (0.37–0.68)	0.66 (0.34–0.79)
SE	0.77 (0.65–0.79)	0.61 (0.37–0.79)	0.63 (0.39–0.73)	0.71 (0.37–0.79)
SWE	0.86 (0.37–0.96)	0.40 (0.10–0.83)	0.36 (0.07–0.66)	0.68 (0.32–0.83)
TWE	0.93 (0.71–0.98)	0.61 (0.37–0.91)	0.57 (0.32–0.71)	0.76 (0.55–0.85)
RWE	0.88 (0.52–0.96)	0.46 (0.16–0.83)	0.43 (0.12–0.62)	0.71 (0.39–0.82)
HHSE	5.63 (4.43–6.26)	4.43 (2.93–6.01)	4.40 (3.02–5.02)	4.81 (3.76–6.03)
ApEn	1.44 (0.63–1.59)	0.95 (0.54–1.35)	1.08 (0.47–1.50)	1.26 (0.63–1.60)
SampEn	1.88 (0.52–2.65)	1.08 (0.15–2.37)	0.97 (0.01–1.63)	1.44 (0.13–2.16)
FuzzyEn	3.28 (1.49–4.33)	1.80 (0.81–4.14)	1.70 (1.01–3.72)	2.22 (1.13–3.44)
SPE	0.81 (0.79–0.83)	0.64 (0.49–0.82)	0.58 (0.46–0.82)	0.65 (0.56–0.75)
TPE	0.91 (0.87–0.92)	0.74 (0.49–0.91)	0.57 (0.44–0.69)	0.62 (0.53–0.80)
RPE	0.91 (0.87–0.92)	0.67 (0.33–0.91)	0.46 (0.29–0.62)	0.60 (0.47–0.79)
MDFA (2)	0.62 (0.23–1.26)	1.67 (0.56–2.25)	1.67 (1.35–2.36)	1.00 (0.72–1.68)
MDFA (−8)	0.54 (0.38–1.32)	1.79 (0.35–2.47)	2.05 (1.54–2.68)	1.43 (0.84–2.06)

RE, response entropy in the M-entropy module; SE, state entropy; SWE, Shannon wavelet entropy; TWE, Tsallis wavelet entropy; RWE, Renyi wavelet entropy; HHSE, Hilbert-Huang spectral entropy; ApEn, approximate entropy; SampEn, sample entropy; FuzzyEn, fuzzy entropy; SPE, Shannon permutation entropy; TPE, Tsallis permutation entropy; RPE, Renyi permutation entropy; MDFA(2), Multifractal detrended fluctuation analysis with q = 2; MDFA(-8), Multifractal detrended fluctuation analysis with q = −8.

To estimate the baseline variability and the sensitivity to the induction process of each index, the CV value of all the indices for the sevoflurane data set are computed and the results are given in Table 2. During the awake state, the CV value of SampEn was 0.095, which was the highest; The CV value of TPE was 0.003, significantly lower than MDFA(2) (0.240) and MDFA(−8) (0.125) and the other indices. The CV values of SPE and RPE were lower than other indices as well. The lower CV value of PE illustrates that PE measures were less sensitive to noise, while MDFA methods were least robust against noise. During induction, the CV of SWE (0.338) was the highest. This demonstrates that SWE had a faster response speed compared to the other indices.

**Table 2:** **The CV of the studied indices at different anesthetic states**.

	**Awake**	**Induction**	**Deep**	**RoC**
RE	0.025	0.149	0.047	0.052
SE	0.016	0.122	0.047	0.050
SWE	0.080	0.338	0.177	0.077
TWE	0.024	0.161	0.063	0.038
RWE	0.043	0.276	0.127	0.057
HHSE	0.029	0.089	0.027	0.024
ApEn	0.040	0.193	0.064	0.043
SampEn	0.095	0.259	0.087	0.094
FuzzyEn	0.089	0.193	0.088	0.073
SPE	0.006	0.115	0.028	0.025
TPE	0.003	0.138	0.030	0.028
RPE	0.004	0.219	0.043	0.041
MDFA(2)	0.240	0.176	0.046	0.100
MDFA(-8)	0.125	0.256	0.047	0.097

**Figure 5:**
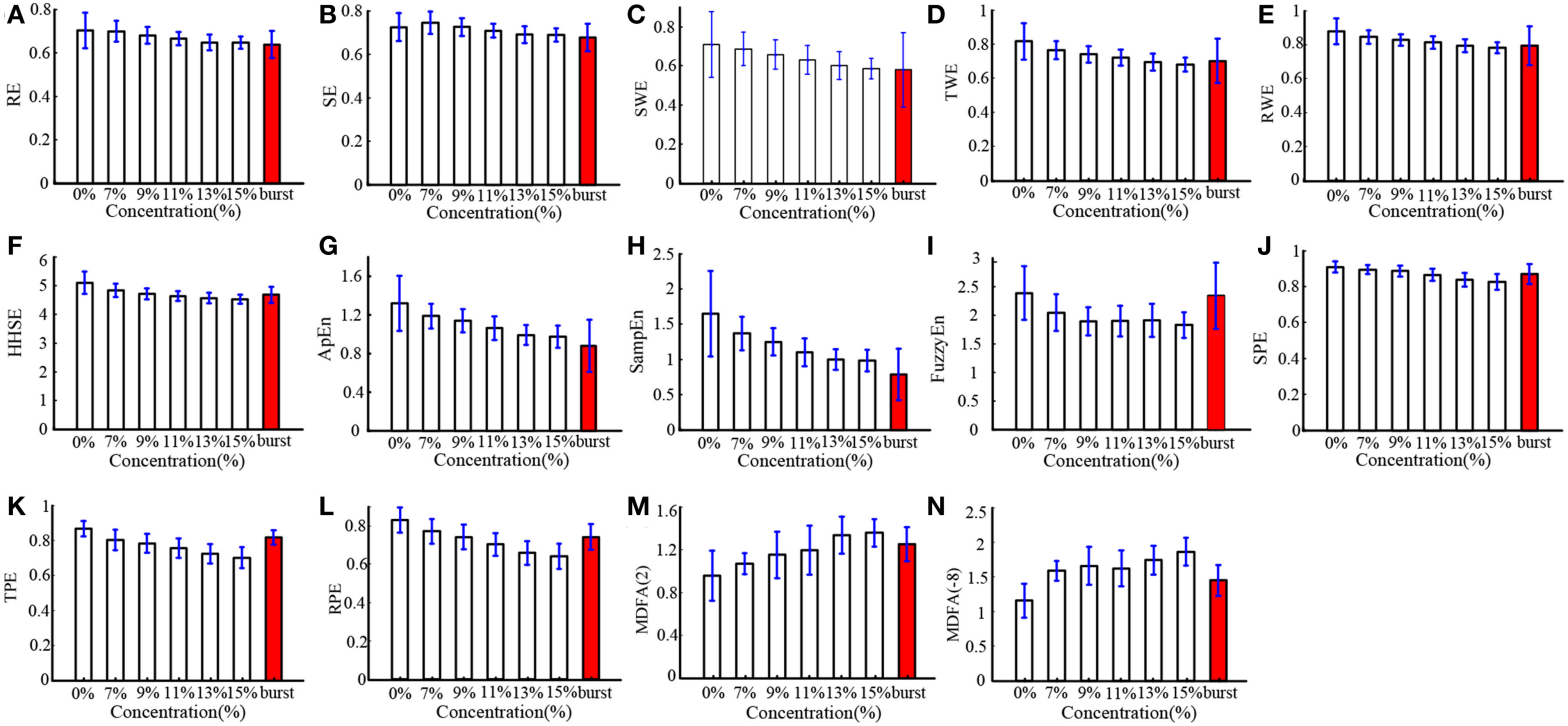
**Histograms of entropy **(A–L)** and MDFA **(M,N)** indices for patients induced with different isoflurane concentrations, including 0, 7, 9, 11, 13, 15% and the concentration at which burst suppression occurred.** The burst suppression state is highlighted by the red bar.

In order to verify the performance of all the indices for monitoring DoA and detecting the burst suppression state, we analyzed the isoflurane anesthesia data set, in which some subjects entered into the burst suppression state during deep anesthesia. The results are given in histogram form and shown in Figure 5. All the indices except SE and MDFA decreased with increasing isoflurane concentration. During burst suppression, only ApEn and SampEn continued to decrease. This means that the ApEn and SampEn algorithms could be used to evaluate DoA including detection of the burst suppression state, without the need for Supplementary Methods. The tabulated results for each index at the different isoflurane concentrations and BSP are presented in Table 3. The CV of the indices show that PE (0.033) outperformed the others in awake state (0% concentration) (see Table 4). And the CV of two MDFA measures were relative higher in awake state. It indicate that MDFA algorithms were no better than some entropy measures in anti-noise performance.

**Table 3:** **The statistics of the studied indices at different isoflurane concentrations [median (min-max)]**.

	**Concentrations and BSP**						
	**0%**	**7%**	**9%**	**11%**	**13%**	**15%**	**BSP**
RE	0.70 (0.42–0.91)	0.70 (0.46–0.80)	0.68 (0.49–0.79)	0.67 (0.50–0.77)	0.65 (0.43–0.73)	0.65 (0.55–0.72)	0.65 (0.37–0.82)
SE	0.73 (0.45–0.83)	0.75 (0.49–0.85)	0.73 (0.52–0.85)	0.71 (0.54–0.83)	0.70 (0.46–0.78)	0.69 (0.59–0.77)	0.69 (0.39–0.83)
SWE	0.73 (0.03–0.95)	0.70 (0.02–0.87)	0.67 (0.30–0.87)	0.63 (0.34–0.81)	0.61 (0.30–0.80)	0.60 (0.41–0.74)	0.62 (0–0.94)
TWE	0.82 (0.24–0.97)	0.76 (0.22–0.91)	0.74 (0.56–0.88)	0.71 (0.14–0.85)	0.70 (0.52–0.86)	0.67 (0.55–0.80)	0.72 (0.12–0.97)
RWE	0.89 (0.36–0.98)	0.85 (0.33–0.95)	0.83 (0.69–0.93)	0.81 (0.24–0.91)	0.80 (0.66–0.91)	0.78 (0.68–0.87)	0.82 (0.19–0.98)
HHSE	5.06 (3.53–5.95)	4.82 (3.57–5.48)	4.71 (3.93–5.33)	4.64 (3.62–5.24)	4.58 (3.66–5.07)	4.53 (4.02–4.95)	4.70 (3.38–5.33)
ApEn	1.45 (0.07–1.60)	1.17 (0.06–1.55)	1.14 (0.82–1.48)	1.06 (0.01–1.42)	0.98 (0.63–1.34)	0.95 (0.73–1.29)	0.90 (0.07–1.51)
SampEn	1.75 (0.03–2.58)	1.31 (0.02–2.18)	1.22 (0.78–1.90)	1.10 (0.01–1.78)	0.99 (0.40–1.49)	0.95 (0.38–1.42)	0.78 (0.02–1.88)
FuzzyEn	2.37 (0.56–3.93)	2.00 (0.33–3.29)	1.86 (1.23–2.89)	1.86 (0.61–3.04)	1.87 (1.13–3.17)	1.81 (1.29–2.65)	2.45 (0.32–3.47)
SPE	0.92 (0.66–0.94)	0.90 (0.39–0.94)	0.89 (0.76–0.94)	0.87 (0.41–0.94)	0.84 (0.69–0.92)	0.82 (0.69–0.92)	0.88 (0.47–0.92)
TPE	0.88 (0.73–0.92)	0.79 (0.65–0.92)	0.78 (0.61–0.91)	0.76 (0.59–0.89)	0.72 (0.59–0.88)	0.69 (0.58–0.85)	0.82 (0.67–0.89)
RPE	0.85 (0.59–0.91)	0.76 (0.60–0.90)	0.74 (0.55–0.90)	0.70 (0.36–0.87)	0.66 (0.47–0.85)	0.63 (0.48–0.81)	0.75 (0.55–0.86)
MDFA (2)	0.96 (0.41–1.61)	1.07 (0.81–1.42)	1.23 (0.56–1.56)	1.20 (0.69–1.66)	1.31 (0.92–1.81)	1.37 (1.01–1.74)	1.27 (0.77–1.95)
MDFA (-8)	1.21 (0.55–2.13)	1.58 (1.19–2.22)	1.69 (1.04–2.32)	1.62 (0.98–2.36)	1.71 (1.09–2.36)	1.88 (1.32–2.59)	1.42 (0.32–2.89)

**Table 4:** **The CV of indices for different isoflurane concentrations**.

	**Concentrations and BSP**						
	**0%**	**7%**	**9%**	**11%**	**13%**	**15%**	**BSP**
RE	0.118	0.070	0.057	0.046	0.056	0.045	0.097
SE	0.089	0.070	0.057	0.046	0.055	0.044	0.093
SWE	0.237	0.125	0.114	0.111	0.118	0.090	0.328
TWE	0.130	0.070	0.064	0.065	0.071	0.060	0.187
RWE	0.087	0.047	0.042	0.045	0.048	0.040	0.143
HHSE	0.077	0.048	0.041	0.037	0.040	0.035	0.060
ApEn	0.216	0.108	0.106	0.114	0.103	0.119	0.308
SampEn	0.368	0.172	0.156	0.178	0.147	0.154	0.466
FuzzyEn	0.196	0.156	0.131	0.141	0.152	0.122	0.249
SPE	0.033	0.028	0.033	0.038	0.046	0.053	0.064
TPE	0.052	0.073	0.069	0.074	0.078	0.085	0.050
RPE	0.079	0.083	0.086	0.086	0.095	0.101	0.090
MDFA(2)	0.24	0.08	0.19	0.19	0.13	0.09	0.13
MDFA(−8)	0.21	0.09	0.17	0.16	0.12	0.11	0.15

**Figure 6:**
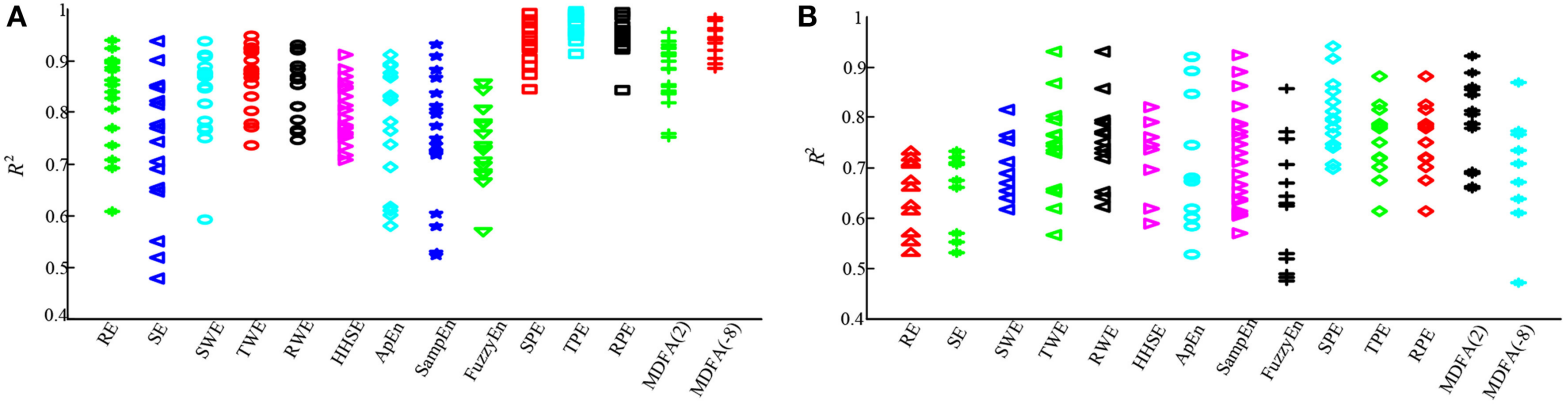
**Statistical analysis of the sevoflurane and isoflurane anesthesia datasets for each of the entropy and MDFA indices. (A)** Maximum coefficient of determination values for sevoflurane anesthesia (*n* = 19). For comparison, the *R*^2^ values for each index are expressed by a different sign and color. **(B)** The *R*^2^ value of the same entropy indices for isoflurane anesthesia (*n* = 20).

To further compare the performance of the studied indices, PK/PD modeling was performed to describe the relationship between the index values and the estimated sevoflurane and isoflurane effect-site concentration. Tables 5, 6 give these parameters for isoflurane and sevoflurane anesthesia respectively, in which the maximum coefficient of determination (*R*^2^) gives the correlation between the index values and the anesthetic effect site concentration. Figures 6A,B show the *R*^2^ values of the indices for the two data sets. Figure 6A shows the *R*^2^ values for sevoflurane. It can be seen that *R*^2^ for TPE (0.95, 95% confidence interval 0.92–0.98) was significantly higher than the other entropy indices. Figure 6B shows *R*^2^ values for isoflurane. Again, *R*^2^ for SPE (0.81) was higher than the other entropy indices. Although *R*^2^ of MDFA with *q* = 8 was relative higher in sevoflurane anesthesia, the value in isoflurane anesthesia was lower. The statistical analysis also shows that for the same entropy algorithm, the mean *R*^2^ value for sevoflurane was significantly higher than for isoflurane.

**Table 5:** **The PK/PD modeling parameters for sevoflurane**.

	***t*_1/2_*k*_eo_(min)**	γ	***E*_max_**	***E*_min_**	**EC_50_**	***R*^2^**
RE	0.04 ± 0.03	8.25 ± 7.62	0.46 ± 0.09	0.13 ± 0.06	1.19 ± 0.60	0.80 ± 0.14
SE	0.06 ± 0.06	5.22 ± 2.32	0.35 ± 0.09	0.14 ± 0.05	1.71 ± 0.93	0.72 ± 0.16
SWE	0.07 ± 0.02	4.01 ± 3.12	1.01 ± 0.16	0.15 ± 0.07	1.42 ± 0.51	0.79 ± 0.12
TWE	0.03 ± 0.01	3.81 ± 1.86	0.50 ± 0.10	0.05 ± 0.16	1.54 ± 0.63	0.86 ± 0.06
RWE	0.04 ± 0.02	5.95 ± 3.98	0.58 ± 0.10	0.12 ± 0.07	1.68 ± 0.60	0.85 ± 0.06
HHSE	0.05 ± 0.02	4.15 ± 3.43	1.99 ± 0.41	0.62 ± 0.34	1.56 ± 1.15	0.80 ± 0.06
ApEn	0.05 ± 0.02	8.22 ± 6.62	0.82 ± 0.17	0.22 ± 0.11	1.84 ± 0.52	0.78 ± 0.11
SampEn	0.05 ± 0.02	5.68 ± 4.45	1.46 ± 0.38	0.40 ± 0.22	1.64 ± 0.62	0.75 ± 0.12
FuzzyEn	0.06 ± 0.04	2.75 ± 1.54	2.14 ± 0.40	0.58 ± 0.32	1.05 ± 0.38	0.69 ± 0.17
SPE	0.70 ± 0.32	4.65 ± 1.57	0.32 ± 0.05	0.08 ± 0.03	1.30 ± 0.33	0.94 ± 0.04
TPE	0.18 ± 0.01	6.98 ± 3.19	0.39 ± 0.04	0.02 ± 0.12	1.33 ± 0.37	0.96 ± 0.02
RPE	0.02 ± 0.01	4.67 ± 3.25	0.50 ± 0.14	0.10 ± 0.16	1.40 ± 0.48	0.95 ± 0.03
MDFA(2)	0.07 ± 0.03	4.92 ± 3.10	0.27 ± 0.15	1.37 ± 0.32	1.52 ± 0.49	0.88 ± 0.06
MDFA(-8)	0.05 ± 0.02	4.54 ± 2.57	0.03 ± 0.27	1.67 ± 0.14	1.33 ± 0.40	0.94 ± 0.03

t_1/2_k_eo_, blood effect-site equilibration constant; γ, slope parameter of the concentration-response relation; E_max_, EEG parameter value corresponding to the maximum drug effect; E_min_, EEG parameter value corresponding to the minimum drug effect; EC_50_, concentration that causes 50% of the maximum effect; R^2^, maximum coefficients of determination.

**Table 6:** **Parameters of PK/PD models for isoflurane**.

	***t*_1/2_*k*_eo_(min)**	γ	***E*_max_**	**E_min_**	***EC*_50_**	***R*^2^**
RE	0.04 ± 0.04	28.88 ± 61.28	0.20 ± 0.04	2.91 ± 0.81	0.91 ± 0.20	0.64 ± 0.07
SE	0.05 ± 0.05	33.32 ± 70.92	0.21 ± 0.04	−1.27 ± 0.50	0.74 ± 0.19	0.65 ± 0.08
SWE	0.05 ± 0.07	19.44 ± 47.62	0.40 ± 0.09	0.14 ± 0.19	1.01 ± 0.20	0.72 ± 0.09
TWE	0.03 ± 0.03	4.80 ± 7.32	0.32 ± 0.11	0.07 ± 0.19	1.00 ± 0.31	0.74 ± 0.09
RWE	0.02 ± 0.01	3.87 ± 6.82	0.23 ± 0.05	0.05 ± 0.15	0.98 ± 0.33	0.75 ± 0.09
HHSE	0.02 ± 0.01	16.70 ± 27.10	1.29 ± 0.58	−5.03 ± 14.83	5.00 ± 10.90	0.72 ± 0.08
ApEn	0.06 ± 0.06	6.46 ± 6.48	0.74 ± 0.27	0.25 ± 0.32	0.75 ± 0.21	0.69 ± 0.17
SampEn	0.03 ± 0.02	5.32 ± 6.73	12.95 ± 13.50	6.79 ± 0.81	0.87 ± 0.28	0.72 ± 0.10
FuzzyEn	0.02 ± 0.01	7.82 ± 15.16	9.21 ± 32.21	0.52 ± 0.42	0.72 ± 0.37	0.61 ± 0.14
SPE	0.06 ± 0.2	3.32 ± 7.35	0.13 ± 0.12	−0.01 ± 0.21	1.30 ± 1.41	0.81 ± 0.07
RPE	0.02 ± 0.01	1.94 ± 5.51	0.42 ± 0.44	0.04 ± 0.34	0.77 ± 0.22	0.78 ± 0.09
TPE	0.01 ± 0.01	5.55 ± 6.64	0.90 ± 2.37	0.08 ± 0.09	0.68 ± 0.24	0.76 ± 0.07
MDFA(2)	0.01 ± 0.02	4.54 ± 10.73	0.17 ± 0.24	0.33 ± 0.45	0.41 ± 0.50	0.78 ± 0.09
MDFA(−8)	0.02 ± 0.01	11.54 ± 20.60	0.02 ± 1.52	1.07 ± 0.51	0.68 ± 0.23	0.69 ± 0.11

**Figure 7:**
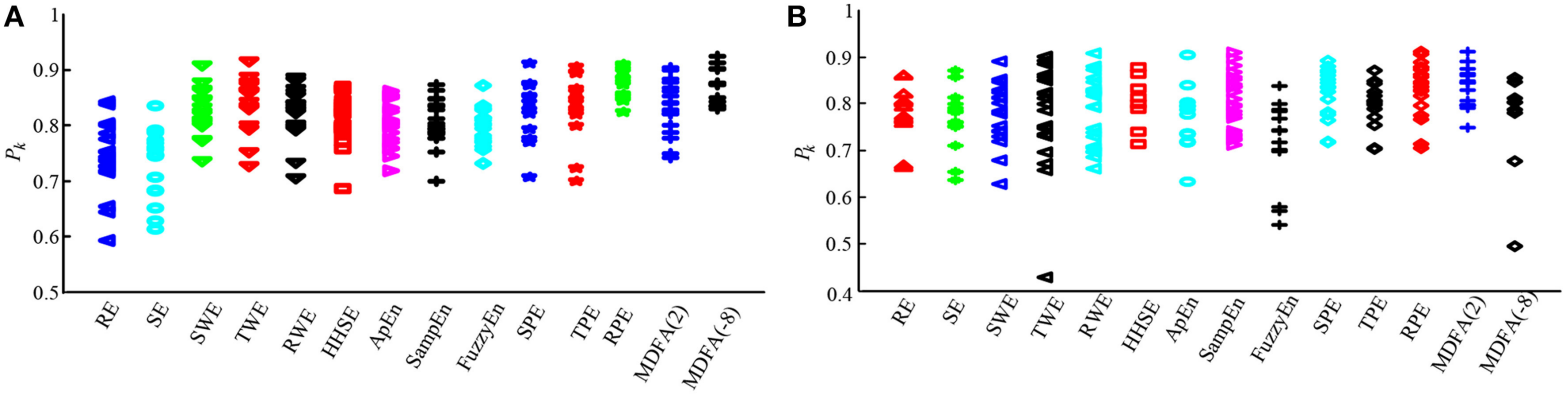
**Statistical analysis of prediction probability (*P*_*k*_) values for sevoflurane and isoflurane anesthesia. (A)** The *P*_*k*_ values for each entropy and MDFA index under sevoflurane anesthesia (*n* = 19). **(B)** The *P*_*k*_ values for each index during isoflurane anesthesia (*n* = 20).

**Figure 8:**
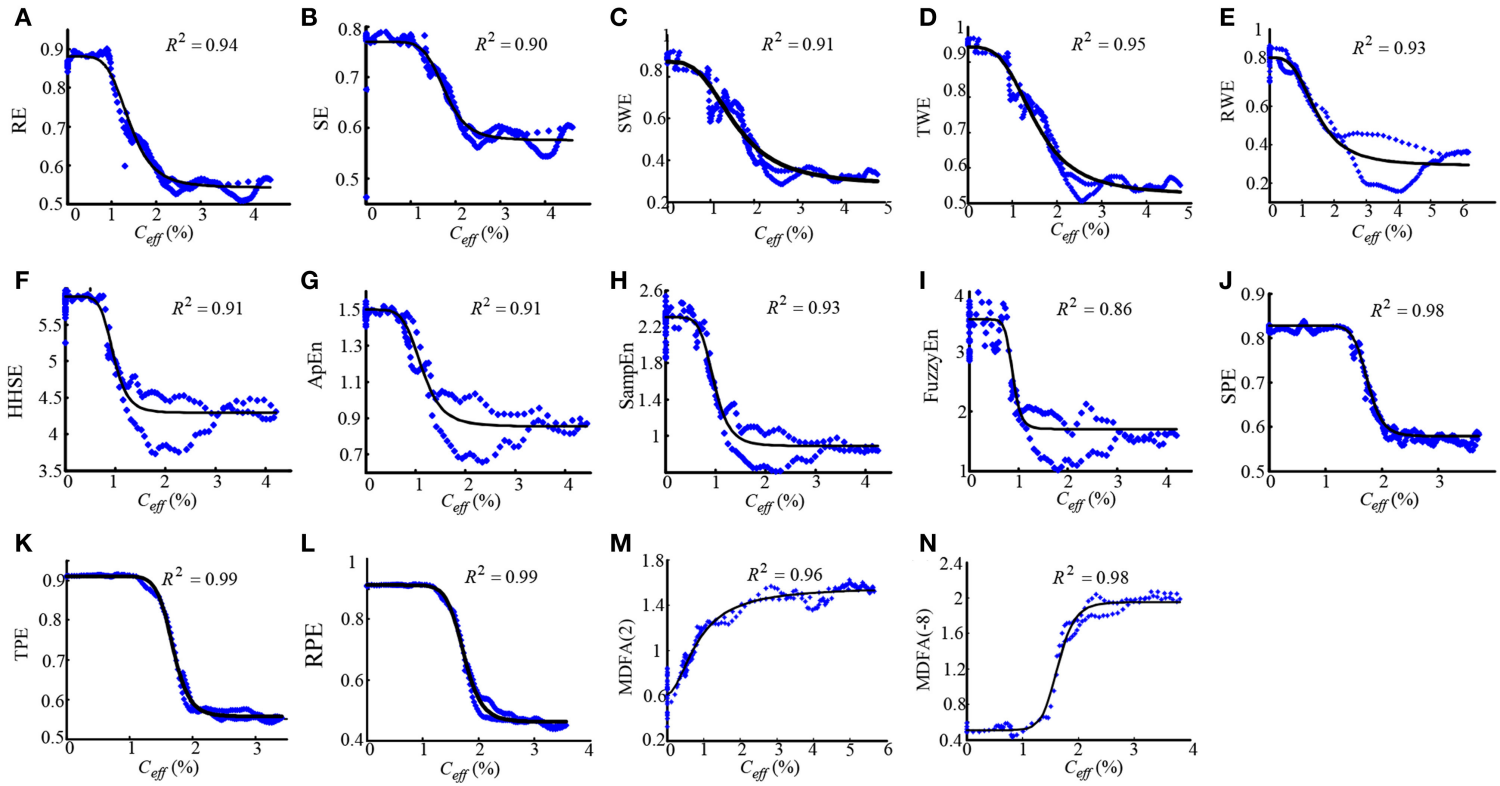
**Dose-response curves between the RE(A), SE(B), SWE(C), TWE(D), RWE(E), HHSE(F), ApEn(G), SampEn(H), FuzzyEn(I), SPE(J), TPE(K), RPE (L), MDFA(2) (M), MDFA(-8) (N) and the sevoflurane *C*_eff_ for the best fit, with the greatest value of *R*^2^ show above the figures.** The dots denote the measured EEG indices values. The solid lines denote the PK/PD modeled EEG index values.

**Figure 9:**
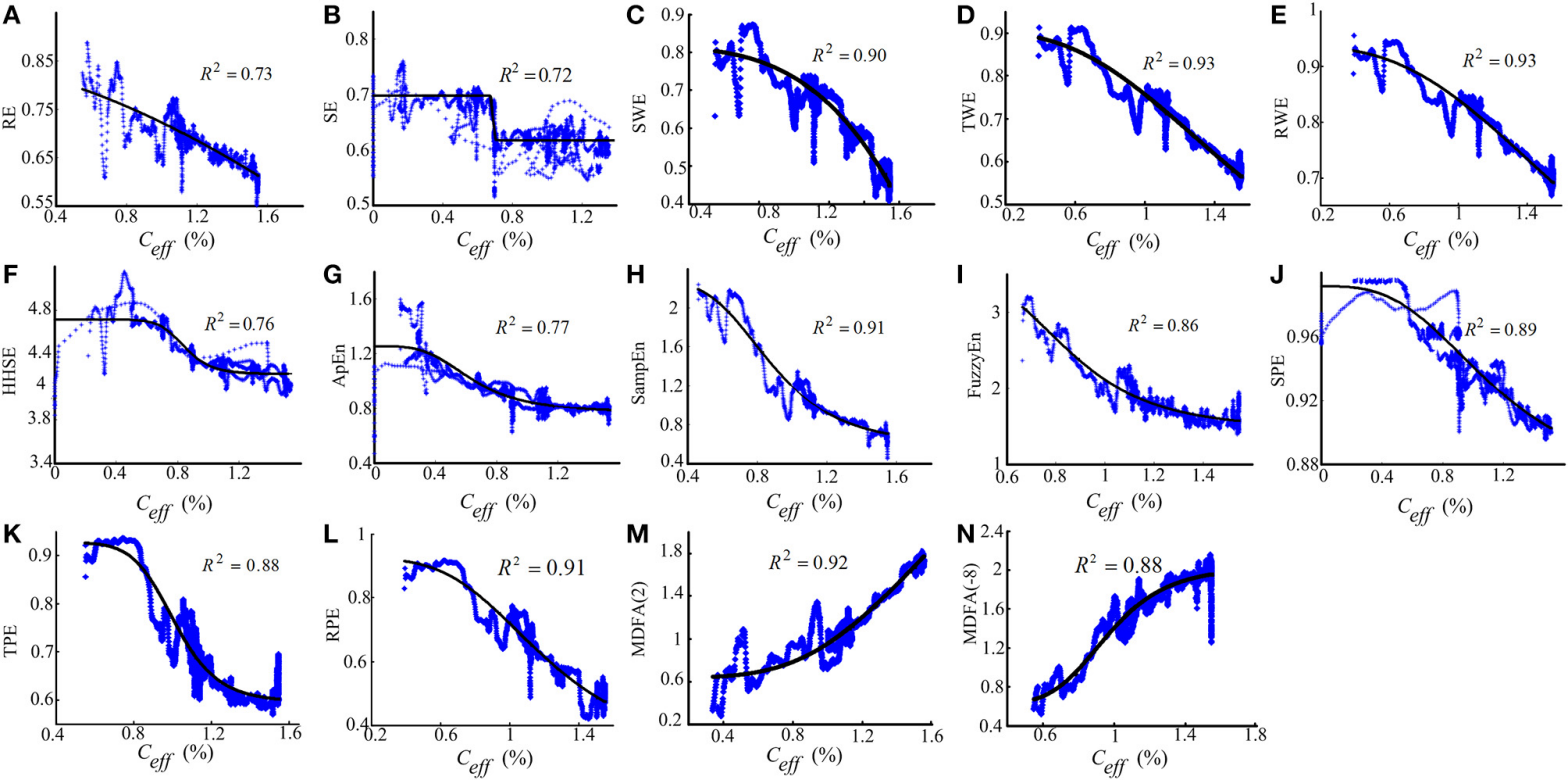
**The similar description as Figure 8 with the dose-response curves between entropy indices and isoflurane effect-site concentration**.

To assess the performance of the indices to correctly predict drug effect-site concentrations, we evaluated the prediction probability *P*_*k*_ of all the indices from the PK/PD modeling for all the subjects, as shown in Figures 7A,B. And the statistical results are shown in Table 7. Overall, most *P*_*k*_ values of indices for sevoflurane were higher than for isoflurane. For sevoflurane, *P*_*k*_ of RPE and MDFA were equal (0.87, 95% confidence interval is 0.83–0.90 and 0.83–0.92 respectively), slightly higher than RWE (0.85) and TWE 0.81 (95% confidence interval 0.79–0.84). Also, *P*_*k*_ of RPE was higher than that of TPE and SPE. Similarly, *P*_*k*_ of RWE was highest in three WE methods. It means that Renyi entropy had a better performance in predicting drug effect-site concentrations comparing with Shannon entropy and Tsallis entropy. The differences between RPE and the other indices were statistically significant (all *p* < 0.05, paired *t*-test), except for MDFA(-8). And the difference between RPE and TPE, SPE were statistically significant (*p* = 0.03 and 0.01 respectively, paired *t*-test), which means that RPE had a stronger ability to track the sevoflurane effect-site concentration during anesthesia. In order to get a more intuitive comparison, the best curve fits of all indices against the effect-site concentration are demonstrated for both sevoflurane (Figure 8) and isoflurane (Figure 9).

**Table 7:** **The *P*_*k*_ statistics for sevoflurane and isoflurane anesthesia for each entropy and MDFA index**.

**Entropy index**	***P*_*k*_ sevoflurane**	***P*_*k*_ isoflurane**
RE	0.74 ± 0.06	0.78 ± 0.06
SE	0.73 ± 0.06	0.77 ± 0.07
SWE	0.83 ± 0.04	0.78 ± 0.07
TWE	0.84 ± 0.05	0.77 ± 0.10
RWE	0.85 ± 0.05	0.78 ± 0.07
HHSE	0.81 ± 0.04	0.80 ± 0.06
ApEn	0.80 ± 0.04	0.77 ± 0.07
SampEn	0.81 ± 0.03	0.81 ± 0.06
FuzzyEn	0.80 ± 0.03	0.71 ± 0.09
SPE	0.83 ± 0.05	0.82 ± 0.05
TPE	0.83 ± 0.06	0.80 ± 0.05
RPE	0.87 ± 0.03	0.83 ± 0.06
MDFA(2)	0.83 ± 0.05	0.83 ± 0.04
MDFA(−8)	0.87 ± 0.03	0.76 ± 0.11

To compare the timeliness performance of each index in tracking DoA, we recorded the computing time of each index for the same subject. 20 EEG recordings from the two data sets were selected. The calculate epoch length (*N*) of each algorithm is equal to 10 s, and the overlap select 5.0 s. The computing time for 1 min of EEG data compared for each index is given in Table 8. The fastest index was WE (0.025 ± 0.001 s). The RE/SE and PE computation times were 0.096 ± 0.008 s and 0.545 ± 0.016 s respectively. The MDFA (16.338 ± 0.280 s) was the slowest. The desktop computer used for this test had the following configuration: Intel Core i3 CPU, 4 cores at 2.93 GHz, with 2 GB of RAM, running Windows XP professional operating system.

**Table 8:** **The computing time for different entropy and MDFA indices for 1 min data length**.

**Entropy index**	**Calculation time(s)**
RE/SE	0.096 ± 0.008
SWE/RWE/TWE	0.025 ± 0.001
HHSE	14.718 ± 1.563
ApEn	2.490 ± 0.098
SampEn	2.541 ± 0.073
FuzzyEn	4.785 ± 0.119
SPE/RPE/TPE	0.545 ± 0.016
MDFA	16.338 ± 0.280

## Discussion and conclusion

In this study, we investigated the performance of 12 entropy algorithms to assess the effect of GABAergic anesthetic agents on EEG activity, including RE, SE, SWE, TWE, RWE, HHSE, ApEn, SampEn, FuzzyEn, SPE, TPE, and RPE. Two data sets including sevoflurane and isoflurane anesthesia were employed as the test samples for evaluating the entropy algorithms. We compared their performance in estimating the DoA and detecting the burst suppression pattern. PK/PD modeling and prediction probability statistics were applied to assess their effectiveness. In addition, we compared the MDFA measure with all entropy indices to test the efficiency of entropy approach.

The twelve entropy measures could be divided into two classes: time-domain-based and time-frequency-domain-based analyses. On one hand, ApEn, SampEn, FuzzyEn, and PE are time domain analysis methods. All these entropy algorithms are based on non-linear theories, and the first three are phase space analytical methods (Chen et al., 2009). PE is based on ordinal pattern analysis of the time series (Bandt, 2005). Considering that the EEG has non-linear characteristics, these four methods have their advantages. For example, FuzzyEn and PE are less sensitive to the signal quality and calculation length (Pincus, 1991; Li et al., 2008a). Relative to ApEn and SampEn, FuzzyEn can resolve more detail in the time series and has more accurate definition in theory (Chen et al., 2009). On the other hand, RE, SE, WE, and HHSE indices are based on the time-frequency domain. The start point of RE and SE is the spectral entropy, which has the particular advantage that the contributions to entropy from any particular frequency range are explicitly separated. In order to achieve optimal response time, RE and SE adopt a variable time window for each particular frequency-called time-frequency balanced spectral entropy (Viertiö-Oja et al., 2004). Compared to the variable time windows of RE and SE, the window function of WE is variable in both time and frequency domains. The HHSE algorithm is based on the EMD and Hilbert transform (Li et al., 2008b). The advantage of this method is that it can estimate the instantaneous amplitude and phase/frequency. Also it can break down a complicated signal without a basis function (such as sine or wavelet functions) into several oscillatory modes that are embedded in this complicated signal. The marginal spectrum gives a more accurate and nearly continuous distribution of EEG energy, which is completely different from the Fourier spectrum (Li et al., 2008b).

Although each entropy algorithm has theoretical advantages with respect to the characterization of EEG recordings during GABAergic anesthesia, we still need to assess the practical performance from several perspectives. In qualitative terms, all the indices are effective at tracking the changes of drug concentration through the EEG analysis. As demonstrated in the presented figures and tables, all the entropies decreased with deepening anesthesia. However, there are quantitative differences between indices for different anesthesia states. This is because the principles underlying each algorithm are entirely different. Entropies based on the time domain, ApEn for example, measure the predictability of future amplitude values of the electroencephalogram based on the knowledge of one or two previous amplitude values. With increasing GABAergic anesthetic drug concentration, the EEG signals become more regular, which leads to a reduction in the ApEn value. Entropies based on the time-frequency domain, such as RE and SE, also decrease with increasing DoA because the EEG shifts to a simpler frequency pattern as the anesthetic dose increases (Rampil, 1998).

In all 12 entropy measures, the TWE, RWE, TPE, and RPE are based on the Tsallis entropy and Renyi entropy theory respectively. Tsallis entropy and Renyi entropy theory are considered generalized concept of entropy compared to Shannon entropy. Similar to Renyi entropy, the Tsallis entropy uses the non-extensive parameter *q* to measure the information of specific events. The results showed that TPE and RPE were better than SPE in assessing the effect of anesthesia. Similar results can also be seen in TWE, RWE, and SWE. There are no studies using TPE or RPE in DoA monitoring before. The excellent performance indicates their potential usefulness in anesthesia analysis.

Furthermore, the coefficient of determination and prediction probability statistics were used to assess the correlation of each index with the anesthetic drug effect site concentration. Three PE measures had a higher *P*_*k*_ and *R*^2^ compared with the other indices. Also, MDFA at *q* = 2 had a relative higher *P*_*k*_ and *R*^2^ in all indices. Comparing anesthetic drugs, the *R*^2^ values for sevoflurane anesthesia were higher than for isoflurane anesthesia, while the *P*_*k*_ values were similar (see Figures 5, 6 and Table 3). This means that the entropy measures were better able to track sevoflurane than isoflurane effect site concentration.

Four additional measures were considered for evaluation of each entropy index. First, the CV was used to evaluate the sensitivity of each index to artifacts during the awake state (Li et al., 2008b, 2010). The results showed that PE outperformed the other indices on this level. In all entropy measures, SWE had the highest CV during anesthesia induction, indicating that this index was superior at discriminating between the awake and anesthetized states. Secondly, the performance for estimating the point of LOC was considered. Although all the entropy measures could distinguish between awake and anesthetized states (see Figure 4), the speed of transition (slope) between the two states was fastest for SWE, while SE had the slowest transition. Thirdly, the performance for discriminating different drug concentrations was considered, especially the ability to distinguish the burst suppression state. The mean ± SD value of the indices showed that all the entropy measures can distinguish different drug concentrations, while only ApEn and SampEn have the ability to distinguish burst suppression from the other states. This means that, if using PE as a DoA index, an additional method for detecting the burst suppression pattern would need to be incorporated, such as Non-linear Energy Operator (NLEO) (Särkelä et al., 2002). The results are in accordance with the findings during desflurane anesthesia for ApEn (Bruhn et al., 2000) and sevoflurane anesthesia for PE and HHSE (Li et al., 2008b, 2010). Finally, the computing time was used to assess algorithm complexity. The results showed that the WE index is the fastest algorithm of all the entropy indices tested. HHSE was the slowest: its computing time for the same data length was about 580 times longer that for WE. In order to improve the computational efficiency, the parallelized method based on the graphics processing unit has been proposed (Chen et al., 2010).

The efficiency of these entropy measures were compared with other two non-linear dynamic measures, the MDFA with *q* = 2 and −8, where MDFA with *q* = 2 is a standard DFA measure. The results and statistics show that MDFA were better in some aspects compared to some of entropy measures, such as sharper slope in LOC, higher *P*_*k*_ and *R*^2^ for sevoflurane (almost equal to RPE) measure. However, there are several shortcomings in MDFA measures. First, CVs of MDFA in awake state were higher compared to those of entropy indices. Second, MDFA could not distinguish the burst suppression state from other states. Most importantly, the computing time of MDFA is the longest in all algorithms, even longer than HHSE, which means that MDFA algorithms are not suitable for real time DoA monitoring. Therefore, entropy approaches are capable for monitoring the EEG changes in anesthesia, and are often advantageous in computation efficiency.

Although this study covers a number of entropy methods and two types of anesthesia, the research has its limitations. For instance, errors caused by individual variability, e.g., age, physical wellness, intraoperative tolerance are hard to control because of the difficulty in data collection in clinical practice. Besides, Interactions between EEG activities and drug concentrations could be studied using finer-grained paradigm, for instance by increasing the drug concentration in a stepwise pattern. Additionally, optimal parameters for each entropy measure may not have been achieved and need further investigation.

This study doesn't provide an absolute measure of “depth” of clinical anesthesia, nor of consciousness for the prevention of intra-operative recall; but rather focuses on understanding the inner workings of each entropy index, and explores whether these indices correlate with GABAergic drug effect. Having a good understanding of the strengths and weaknesses of each measure is necessary before possibly applying them within a clinical context.

In conclusion, each entropy measure has its advantages, and several indices show promise as a simple open-source method for quantifying the brain effects of GABAergic drugs. In particular, the PE indices perform better than other entropy indices as an EEG derivative in several aspects, especially for RPE measure. However, further work is required to accurately quantify the burst suppression pattern. Also, to be useful as a clinical measure, each algorithm still needs additional parameter and computation efficiency optimizations.

### Conflict of interest statement

The authors declare that the research was conducted in the absence of any commercial or financial relationships that could be construed as a potential conflict of interest.
