# Supplementary material for: EEG entropy measures in anesthesia
Source: Front Comput Neurosci. 2015 Feb 18;9:16. doi: 10.3389/fncom.2015.00016 (PMC4332344; doi:10.3389/fncom.2015.00016)
Supplement: Supplementary file 1 [file DataSheet1.DOCX]

# APPENDIX

Each entropy index contains several parameters, which can severely impact the output of its index. Therefore, it is very important to select the appropriate parameters. In anesthesia researches, there are many methods to select parameters including the inter-individual variations, e.g. the relationship with drug effect-site concentration obtained from PK/PD and prediction probability ([Bruhn et al., 2000](#_ENREF_10);[Li et al., 2012](#_ENREF_30)). In this study, the method of selection the parameters is based on indices’ performance in distinguishing different anesthesia states and inter-individual variations. 50 data sets in awake, deep anesthesia and recovery state from 19 patients are selected respectively. The RE and SE algorithms applied in the study adopts the Datex-Ohmeda S/5TM entropy module ([Viertiö‐Oja et al., 2004](#_ENREF_61)). The PE’s parameter selection are based on our previous work ([Li et al., 2010](#_ENREF_29);[Li et al., 2012](#_ENREF_30)). The details of other entropy algorithm’s parameters are discussed as follows. All the results are given by mean±standard deviation. The blue, red and green color represent the awake state, deep anesthesia state and recovery state respectively.

**(A) WE**. There are three types of WE measures (SWE, TWE, RWE) considered in this study. The parameters include basis functions, data length $N$, Tsallis entropy parameter $q$ and Renyi entropy parameter $a$. The basis functions and data length selection based on the SWE. First, several common basis functions including Haar, Dubechies, Coiflets, Sysmlets and Biorthgonal wavelet families were selected. The result is shown in figure S1(a-e). $N$ is assumed to be 1000. As can be seen, only the SWE based on the Biorthgonal basis function can completely separate anesthesia state (red color) from awake (blue color) and recovery states (green color), without overlap. However, there are some basic functions in the Biorthgonal family. Figure S1 (e-g) show the results obtained by bior2.2, bior3.3, bior4.4. It can be seen that the SWE achieved by bior3.3 not only distinguishes anesthesia states from non-anesthesia, but also differentiate between wake and recovery states, especially when the number of layers is 2 and 3. So in this study, the bior3.3 was chosen as the wavelet basis function, and the number of layers was 3. Then, based on them, the selection of $N$ is given in Figure S1 (h). $N$ ranges from 500 to 3000 points with the step of 500 points under the sample of 100Hz. The figure shows that when $N$ ≥ 1000, there is no significant difference in WE for each state. So $N$=1000(10 s) was selected to calculate the WE. Furthermore, based on the parameters of basis function and $N$, the selections of $q$ in TWE and $a$ in RWE are given in Figure S1 (i-j) and Figure S1(k-l).

Figure S1. The changes of WE in different anesthesia with different parameters. (a-g) The change of SWE with different wavelet basis functions, (a) haar; (b) db2; (c) coif2; (d)sysm2; (e) bior2.2; (f) bior3.3; (g) bior4.4. The horizontal axis shows the number of layers(n) decomposed by corresponding wavelet function. The vertical axis shows SWE values. (h) The changes of SWE with different N. (i-j) The changes of TWE with $0<q<1$(i) and $q>1$(j). (k-l) The changes of RWE with $0<a<1$(k) and $a>1$(l).

**(B) HHSE**. In order to choose an appropriate data length N in HHSE algorithm at the sample rate of 100 Hz, A series of $N$ were used to calculate the HHSE in different anesthesia states. The result is shown in figure S2. All values of $N$ could distinguish different anesthesia states. And when the data length was equal to or greater than 1000, the HHSE value would be nearly invariable with the changes of N. So $N$=1000(10 s) was selected in this study.


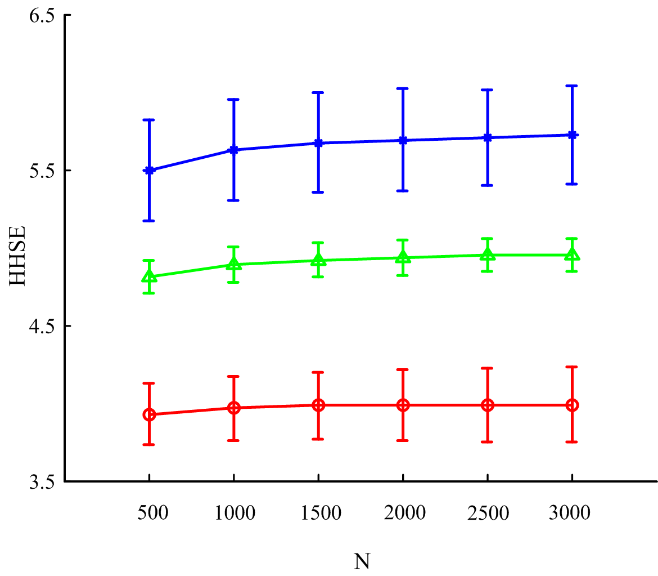


Figure S2. The change of HHSE with different $N$at different anesthesia states. $N$ is from 500 to 3000 stepped by 500 points.

**(C) ApEn**. Figure S3 (a-b) respectively give the result of ApEn over different $r$ in different anesthesia states with $N$=1000, $m$=2 and $m$=3. With increasing $r$, the ApEn values in awake state and recovery state increased and then decreased, while it monotonously changes with r in deep anesthesia state. Both figures show that when $r$ is 0.2 or 0.25 of SD, the difference between deep anesthesia and other states is larger. Considering that the $r$ in ApEn is also used to suppress the noise, its value is chosen as small as possible. The $r$ was chosen as 0.2 of SD. Figure S3 (c) shows the selection of m with $N$=1000 and $r$=0.2*SD. It can be seen the ApEn nearly doesn’t change with $m$. Meanwhile when $m$ is 2, the inter-individual variations of ShEn is smaller. With $r$=0.2*SD and $m$=2, the selection of $N$ is shown in figure S3 (d). The ApEn was very dependent on $N$ and it increased with enlarging $N$, as well as its inter-individual variations, especially in awake and recovery states. The difference between awake and anesthesia state also became larger, but when $N$ was greater than 1000, the difference was not obvious. Therefore, $r$=0.2*SD, $m$=2 and $N$=1000 was selected in this study, which is consistent with the study ([Bruhn et al., 2000](#_ENREF_10)) through different method to choose parameter was used.


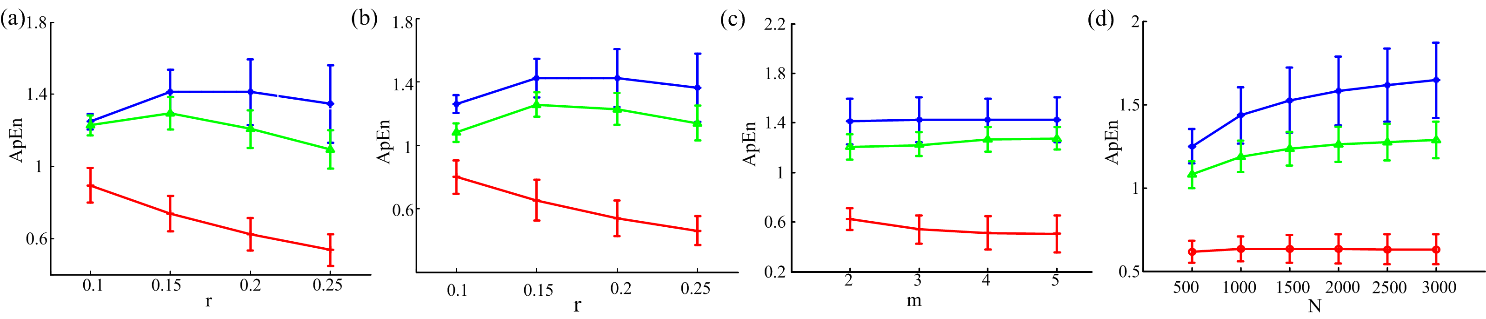


Figure S3. The changes of ApEn with different parameters at different anesthesia states. (a) $m$=2, $N$=1000; (b) $m$=3, $N$=1000;(c) $r$=0.2*SD, $N$=1000; (c) $r$=0.2*SD, $m$=2.

**(D) SampEn.** Figure S4 (a-b) respectively show the changes of SampEn over different $r$ in different anesthesia states, with $N$=1000, $m$=2 and 3. The SampEn values monotonously decreases with increasing $r$ in all states. The difference between awake state and deep anesthesia was obvious, but not between awake and recovery state. There is much overlap between them. Considering the inter-individual variations, $r$=0.2 is better. Figure S4(c) shows the changes of SampEn over different m with $r$=0.2, $N$=1000. There is no significant difference in different $m$ values. For simplicity, $m$=2 was selected. The selection of $N$ is given in figure S4 (d). The SampEn values with $r$=0.2, $m$=2 were almost invariable as $N$ increased. This implies that SampEn values are not dependent on $N$. Finally $N$=1000 (10 s at the sample rate of 100 Hz), $m$=2, $r$=0.2 was selected in this study.


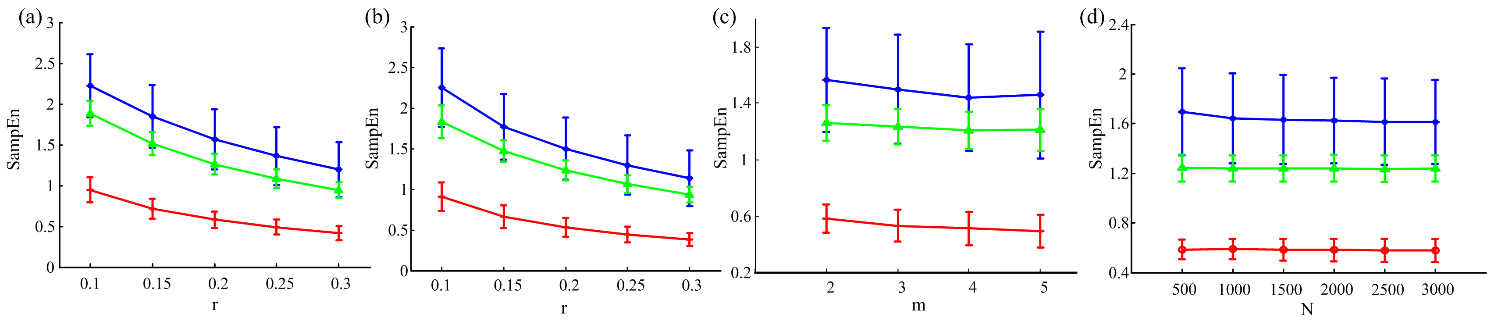


Figure S4. The changes of SampEn with different parameters at different anesthesia states. (a) $m$=2, $N$=1000; (b) $m$=3, $N$=1000; (c) $r$=0.2*SD, $N$=1000; (c) $r$=0.2*SD, $m$=2.

**(E) FuzzyEn.** Figure S5 gives the changes of FuzzyEn with different parameters in different anesthesia states. Accordingly, we selected $r$=0.2, $m$=2 and $N$=1000 for the computation of FuzzyEn in this study.


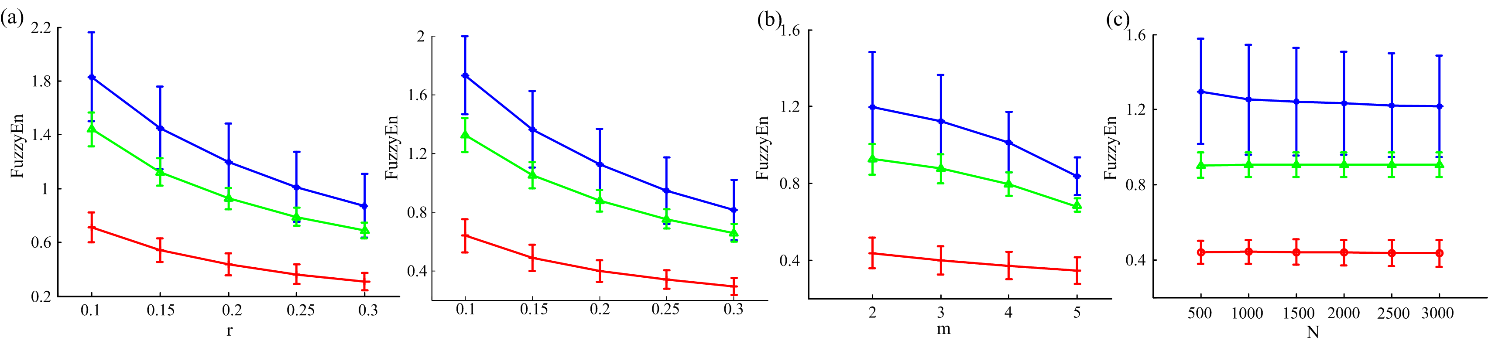


Figure S5. The changes of FuzzyEn with different parameters at different anesthesia states. (a) $m$=2, $N$=1000; (b) $m$=3, $N$=1000;(c) $r$=0.2*SD, $N$=1000; (c) $r$=0.2*SD, $m$=2.

**(F) TPE and RPE.** The embedded dimension $m$ , lag $\tau$ and data length $N$ had been discussed in our previous study ([Li et al., 2008a](#_ENREF_31);[Li et al., 2010](#_ENREF_29);[Li et al., 2012](#_ENREF_30)). It is suggested that $m=6$ and $\tau=1$ are suitable for the sevoflurane DoA monitoring and $m=3$ ,$\tau=2$ is better for isoflurane analysis. So, for the parameters of TPE and WPE, we only considered the embedded dimension of 3 and 6. For the sevolurane, using $\tau=1$ and isoflurane is $\tau=2$. Figure S6 (a-b) are the TPE of three anesthesia states at the $0<q<1$ and $q>1$ respectively, the $m=3$. Figure S6 (c-d) shows the TPE of $m=6$ at $0<q<1$ and $q>1$ respectively. It can be seen that $m=6$, $q=0.9$ has a better performance in TPE. Figure S6 (e-h) are the RPE measure of three anesthesia states similar as Figure S (a-d). We select the $m=6$ and $a=2$ for RPE calculation.

Figure S6. The changes of TPE and RPE with different parameters at different anesthesia states. (a-d) The TPE measures with different parameters: (a) $m=3$,$0<q<1$; (b) $m=3$, $q>1$; (c) $m=6$, $0<q<1$; (d) $m=6$, $q>1$. (e-h) The RPE measures with different parameters: (e) $m=3$,$0<a<1$; (f) $m=3$, $a>1$; (g) $m=6$, $0<a<1$; (h) $m=6$, $a>1$.

**(G) MDFA.** In order to choose an appropriate $q$ in MDFA algorithm, a series of $q$ were used to calculate the MFDFA in different anesthesia states. The result is shown in Figure S7. All values of $q$ could distinguish different anesthesia states. The MDFA values monotonously decrease with increasing $q$ in recovery state. We choose $q=-8$ because when $q$ is -8, the difference between deep anesthesia and other states is larger, and the inter-individual variations of MDFA is smaller. Meanwhile, we also choose $q=2$ for its better performance while it becomes a standard DFA measure.

Figure S7. The change of MDFA with different $q$ at different anesthesia states. The parameter $q$ is from -10 to 10 stepped by 2.
